# Naa10p promotes cell invasiveness of esophageal cancer by coordinating the c-Myc and PAI1 regulatory axis

**DOI:** 10.1038/s41419-022-05441-0

**Published:** 2022-11-24

**Authors:** Ke-Fan Pan, Yu-Cheng Liu, Michael Hsiao, Tsu-Yao Cheng, Kuo-Tai Hua

**Affiliations:** 1 Graduate Institute of Toxicology, College of Medicine, National Taiwan University, Taipei, Taiwan; 2 Department of Medical Education and Research, Wan Fang Hospital, Taipei Medical University, Taipei, Taiwan; 3 Division of General Surgery, Department of Surgery, Wan Fang Hospital, Taipei Medical University, Taipei, Taiwan; 4 Division of Colorectal Surgery, Department of Surgery, Wan Fang Hospital, Taipei Medical University, Taipei, Taiwan; 5 Genomics Research Center, Academia Sinica, Taipei, Taiwan; 6 Department of Laboratory Medicine, National Taiwan University Cancer Center and National Taiwan University College of Medicine, Taipei, Taiwan; 7 Division of Gastroenterology, Department of Internal Medicine, National Taiwan University Hospital and National Taiwan University College of Medicine, Taipei, Taiwan

**Keywords:** Oncogenes, Tumour biomarkers, Acetylation, Metastasis, Cell signalling

## Abstract

N-α-acetyltransferase 10 protein, Naa10p, is involved in various cellular functions impacting tumor progression. Due to its capacity to acetylate a large spectrum of proteins, both oncogenic and tumor-suppressive roles of Naa10p have been documented. Here, we report an oncogenic role of Naa10p in promoting metastasis of esophageal cancer. *NAA10* is more highly expressed in esophageal cancer tissues compared to normal tissues. Higher *NAA10* expression also correlates with poorer survival of esophageal cancer patients. We found that *NAA10* expression was transcriptionally regulated by the critical oncogene c-Myc in esophageal cancer. Furthermore, activation of the c-Myc-Naa10p axis resulted in upregulated cell invasiveness of esophageal cancer. This increased cell invasiveness was also elucidated to depend on the enzymatic activity of Naa10p. Moreover, Naa10p cooperated with Naa15p to interact with the protease inhibitor, PAI1, and prevent its secretion. This inhibition of PAI1 secretion may derive from the N-terminal acetylation effect of the Naa10p/Naa15p complex. Our results establish the significance of Naa10p in driving metastasis in esophageal cancer by coordinating the c-Myc-PAI1 axis, with implications for its potential use as a prognostic biomarker and therapeutic target for esophageal cancer.

## Introduction

Esophageal cancer (ESCA) is an aggressive malignancy with poor therapeutic outcomes [1]. Alterations in proteins and posttranslational modifications (PTMs) leading to the pathogenesis of ESCA have attracted much research attention [2, 3]. Since large-scale genome profiling has identified several driver mutations and key regulation pathways but effective targeted therapy is still lacking, discovering novel pathological alterations in proteins may give rise to advances in drug development to fulfill the gap in current drug targets. A variety of PTMs such as phosphorylation, acetylation, and other novel modifications have become increasingly appreciated in the process of tumor progression [4, 5]. Therefore, targeted therapies which inhibit critical modifications of ESCA have been developed. For example, tyrosine kinase inhibitors (TKIs) have been reported to improve the prognosis of ESCA patients by utilizing them alone or in combination with traditional therapies [6]. It has been established that these TKIs of oncogenes, including EGFR, HER2, VEGFR, and MET, block the binding of the ligands to the receptor and inhibit intracellular tyrosine kinase activity [7–9]. Protein acetylation has also been addressed as a potential novel therapeutic drug target to repress cancer cell proliferation. In particular, histone deacetylase (HDAC) inhibitors and histone acetyltransferase (HAT) inhibitors are proposed to improve ESCA patients’ survival by arresting the cell cycle, inhibiting differentiation, suppressing angiogenesis, and inducing apoptosis [10]. However, targeting N-α-terminal acetylation, which is the most common modification, occurring in ~85% of human proteins, still suffers from numerous hurdles and uncertainties in the efficacy of clinical practice [11, 12]. Hence, addressing such challenges and discovering novel therapeutic strategies to alter the modifications that occur in the N-terminus of proteins may improve treatment options for patients with ESCA.

N-α-terminal acetylation has been reported to affect the lifespan, folding characteristics, and binding properties of the acetylated protein. By appending an acetyl group to the N-terminal amino group, the charge, hydrophobicity, and size of the N-terminus are altered irreversibly. In this process, N-terminal acetyltransferases (NATs) are prominent players which co-translationally or post-translationally affect the N-terminal extremity of most proteins [12, 13]. A total of eight NATs are currently known and the major modifiers of the human proteome are NatA and NatB, which have been estimated to N-α-acetylate 38% and 21% of all proteins respectively [12]. The human NatA complex, which consists of the catalytic subunit N-α-acetyltransferase 10 protein (Naa10p), the ribosomal anchor Naa15p, and the auxiliary subunit HYPK, co-translationally acetylates N-termini that bear small amino acids [14, 15]. Notably, Naa10p also exists in a monomeric state with the capacity to post-translationally acetylate acidic N-termini in vitro, but this effect does not appear to have activity towards classical NatA-type substrates [14, 16]. Since NatA is estimated to acetylate such a high number of proteins in the human proteome, it is not surprising that Naa10p has been implicated in numerous diseases, including cancers and developmental disorders [17, 18]. Several reports have linked dysregulated Naa10p expression to various human cancers, such as breast, prostate, lung, liver, cervical, bladder, and colorectal cancers [19–21]. However, possibly due to its capacity to acetylate a large spectrum of target proteins, both tumor-suppressive and oncogenic roles have been documented [17]. For example, high Naa10p expression was reported to correlate with a favorable prognosis for lung, breast, and oral cancer patients [22–24]. In contrast, correlations between Naa10p expression and poor survival outcomes were reported in prostate, liver, bone, and colorectal cancer [21, 25, 26].

In this study, we aimed to investigate the impact of Naa10p on ESCA progression. Higher *NAA10* expression was observed in ESCA tumor tissues compared to normal tissues. We also found a prognostic value of *NAA10* in ESCA. Our functional analysis showed a critical and specific role of Naa10p in enhancing the invasiveness of ESCA cells. Molecular mechanisms underlying the upstream and downstream regulations of Naa10p are deciphered, as well as the impact of their regulatory axis on the metastatic properties of tumor cells.

## Materials and methods

### DNA constructions, antibodies, cell culture, and transfection

Plasmid bearing *NAA10* construct was engineered by PCR and subcloned into a pWPI vector. The point mutation in the acetyltransferase domain of *NAA10* (R82A) was obtained using the QuikChange® mutagenesis kit (Stratagene, La Jolla).

The following antibodies were used: Naa10p (GB-10511, Genesis biotech, Taiwan) for immunoblotting, c-Myc (ab32072, Abcam) for immunoblotting, Naa15 (sc365931, Santa Cruz) for immunoblotting and immunoprecipitation, Cystatin E/M (AF-1286, R&D) for immunoblotting, PAI1 (AF-1786, R&D) for immunoblotting, PAI1 (ab222754, Abcam) for immunoprecipitation, TIMP2 (AF-971, R&D) for immunoblotting, V5 (ab9116, Abcam) for immunoblotting and immunoprecipitation, Acetylated lysine (9681, Cell Signaling) for immunoblotting, Acetylated lysine (05-515, Sigma) for immunoprecipitation.

Human esophageal cancer cell lines KYSE50, KYSE70, KYSE170, and KYSE510 were kindly provided by Dr. Michael Hsiao (Genomic Research Center, Academia Sinica, Taiwan). KYSE50, KYSE70, KYSE170, and KYSE510 cells were grown in RPMI-1640 medium supplemented with 1% penicillin-streptomycin and 10% fetal bovine serum at 37 °C with 5% CO_2_. All the cell lines were tested for mycoplasma contamination.

### ShRNA sequences

The shRNA constructs against *NAA10*, *NAA15*, *MYC*, *CST6*, *SERPINE1*, and *TIMP2* were purchased from National RNAi Core Facility (Academia Sinica, Taiwan). The target sequences are as follows:

shNAA10-1: 5′-CCAGATGAAATACTACTTCTA-3′;

shNAA10-2: 5′-CCACGAGCTTTCACAATAAATT-3′;

shNAA15-1: 5′-TTGGACCATATCTAGTATATA-3′;

shNAA15-2: 5′-GCCATTAAGTGTTACAGAAAT-3′;

shMYC-1: 5′-GAATGTCAAGAGGCGAACACA-3′;

shMYC-2: 5′-CCTGAGACAGATCAGCAACAA-3′;

shCST6-1: 5′-GCTGCGCTGTGACTTTGAGGT-3′;

shCST6-2: 5′-CCGAGACACGCACATCATCAA-3′;

shSERPINE1-1: 5′-TCTCTGCCCTCACCAACATTC-3′;

shSERPINE1-2: 5′-GTGCCTGGTAGAAACTATTTC-3′;

shTIMP2-1: 5′-CAAGTTCTTCGCCTGCATCAA-3′;

shTIMP2-2: 5′-CCTGAGAAGGATATAGAGTTT-3′;

### Lentivirus production and infection

To prepare lentiviral particles, 293T human embryonic kidneys were transfected using calcium phosphate transfection. Briefly, 293T cells were cultured in 10-cm^2^ dishes one day before transfection. Then, the cells were transfected with 10 μg DNA together with 10 μg of pCMVΔR8.91 (packaging vector) and 1 μg of pMD.G (envelope vector). After 16 h of incubation, the transfection medium was replaced with fresh culture medium. Forty-eight hours later, the lentivirus-containing medium was collected from transfection and spun down at 1500 rpm for 5 min to pellet the cell debris; the supernatant was filtered with a 0.45 μm filter, and the target cells, cells were infected with the lentivirus containing medium (supplemented with 8 μg/ml polybrene) for 48 h.

### Gene expression analysis from public databases

Overall survival analysis of ESCA patients from the TCGA-ESCA database was performed using the Kaplan–Meier method. Patients were subsequently subgrouped based on their relative expression of *NAA10*, *NAA15*, *MYC*, and *SERPINE1*. For the gene expression analysis, we used three datasets (the TCGA-ESCA, GSE26886, and GSE75241). Patients were subsequently subgrouped based on their tumor subtypes.

### Gene-set enrichment analysis

Publicly available TCGA gene expression data of ESCA samples were downloaded from the UCSC Xena Platform. These cases were subgrouped into *NAA10*-high and *NAA10*-low based on their relative expression of *NAA10*, and GSEA was performed using Gene Ontology gene sets.

### qRT-PCR

Cellular total RNA was isolated with NucleoZOL reagent (Macherey-Nagel). The cDNA was synthesized with the PrimerScript RT reagent kit (Takara). The real-time PCR reaction was performed with iTaq Universal SYBR Green Supermix using the CFX Connect Real-Time PCR System according to the manufacturer’s protocol (Bio-Rad). The fluorescence data of the detected genes were normalized to the expression of actin using the 2-ΔΔCT method. The primers utilized in our investigation are listed in Table S2.

### Immunoblotting

Cells were harvested and lysed in RIPA buffer and heated at 95 °C for 10 min. Protein samples were separated by SDS-PAGE, transferred to a polyvinylidene difluoride membrane (PVDF), blocked and incubated with the indicated primary antibody and horseradish peroxidase (HRP)-conjugated secondary antibody. The immune complexes for western blotting were visualized using an enhanced ECL system.

### Luciferase reporter assay

Cells were seeded and transfected with the *NAA10*-Luc reporter vector together with a Renilla luciferase plasmid at a ratio of 10:1. The luciferase activity of the cells was analyzed with the Dual-Luciferase® Reporter Assay System according to the manufacturer’s instructions (Promega). The relative levels of luciferase activity were normalized to the Renilla luciferase activity levels.

### Cell proliferation assay

Cells were seeded at a density of 1 × 10^3^ cells per well in 96-well dishes and incubated at 37 °C for the indicated number of days. The cell viability index was determined by the Cell Counting Kit-8 according to the manufacturer’s instructions.

### Colony formation assay

Cells were seeded at a density of 1 × 10^3^ cells per well in six-well dishes and incubated at 37 °C for 10 days. Cells were fixed and stained with crystal violet 0.2%/ methanol 20%. Quantification was performed by counting the stained cells.

### Two-chamber migratory assay

Cell migration ability was determined using a modified two-chamber invasion assay (8 μm pore size, Merck Millipore) according to the manufacturer’s instructions. About 5 × 10^4^ cells were seeded into the upper chamber and the cells were allowed to invade into the lower chamber for 24 h. Cells in the upper chamber were carefully removed using cotton buds and cells at the bottom of the membrane were fixed and stained with crystal violet 0.2%/methanol 20%. Quantification was performed by counting the stained cells.

### Two-chamber invasion assay

Cell invasion ability was determined using a modified two-chamber invasion assay (8 μm pore size, Merck Millipore) according to the manufacturer’s instructions. About 1 × 10^5^ cells were seeded into the upper chamber which was coated with 40 μl Matrigel and the cells were allowed to invade into the lower chamber for 48 h. Cells in the upper chamber were carefully removed using cotton buds and cells at the bottom of the membrane were fixed and stained with crystal violet 0.2%/methanol 20%. Quantification was performed by counting the stained cells.

### In vivo subcutaneous metastasis mouse model

Two groups of six male NOD-SCIDγ mice were injected with KYSE70-shCtl cells and KYSE70-shNAA10 cells. Tumor cells were suspended in PBS with 50% Matrigel on ice before the xenograft procedure. After inoculation for 5 weeks, all mice were then euthanized. The tumors were collected, weighted, and photographed at the end of the experiments. The lungs of these mice were also removed and luciferase activity in the excised organs (measured in photons) was determined using an IVIS Spectrum imaging system (the Xenogen IVIS® Spectrum system in the Animal Center, NTU). All animal procedures were using protocols approved by the National Taiwan University College of Medicine and College of Public Health Institutional Animal Care and Use Committee, with the IACUC Approval number: 20180350.

### Human protease and protease inhibitor array analysis

Cells were harvested and lysed in RIPA buffer. Cell culture-conditioned medium was also collected. Cell lysates (200 μg) or conditioned medium (500 μL) with 15 μL protease/protease inhibitor antibody cocktails were incubated in a total volume 1.5 mL of array buffer 6 at room temperature for an hour. The mixtures were then incubated with array membranes at 4 °C for 18 h. The membranes were washed and incubated with diluted streptavidin-HRP at room temperature for 30 min. The membranes were then incubated with ChemiReagent Mix and exposed to X-ray film for 1–10 min. The data were analyzed by Image J.

### Immunoprecipitation

Cells were lysed on ice for 30 min in immunoprecipitation buffer (0.5% NP-40, 20 mM Tri-HCl, 150 mM NaCl, and 1 mM EDTA) containing protease inhibitor cocktail. Protein concentration was determined by the BCA assay. Cell lysates (1 mg) were incubated with 1 μg of indicated antibodies or control IgG at 4 °C overnight with rotary agitation. Protein A/G-Sepharose beads were then added to the lysates and incubated for another 2 h. Beads were washed five times with ice-cold immunoprecipitation buffer and boiled for 10 min in 1x sample loading buffer. Whole-cell lysates and immunoprecipitants were resolved by SDS-PAGE and analyzed by western blotting.

### Statistical analysis

All data were statistically analyzed with GraphPad Prism 5 and Sigma Plot 10.0 software. A two-tailed *t* test was utilized to analyze the difference between two groups. Data were presented as mean ± SD. All findings were considered significant at a *P* value threshold of <0.05.

## Results

### NAA10 overexpression was found in both esophageal adenocarcinoma (EAC) and esophageal squamous cell carcinoma (ESCC)

**Fig. 1:**
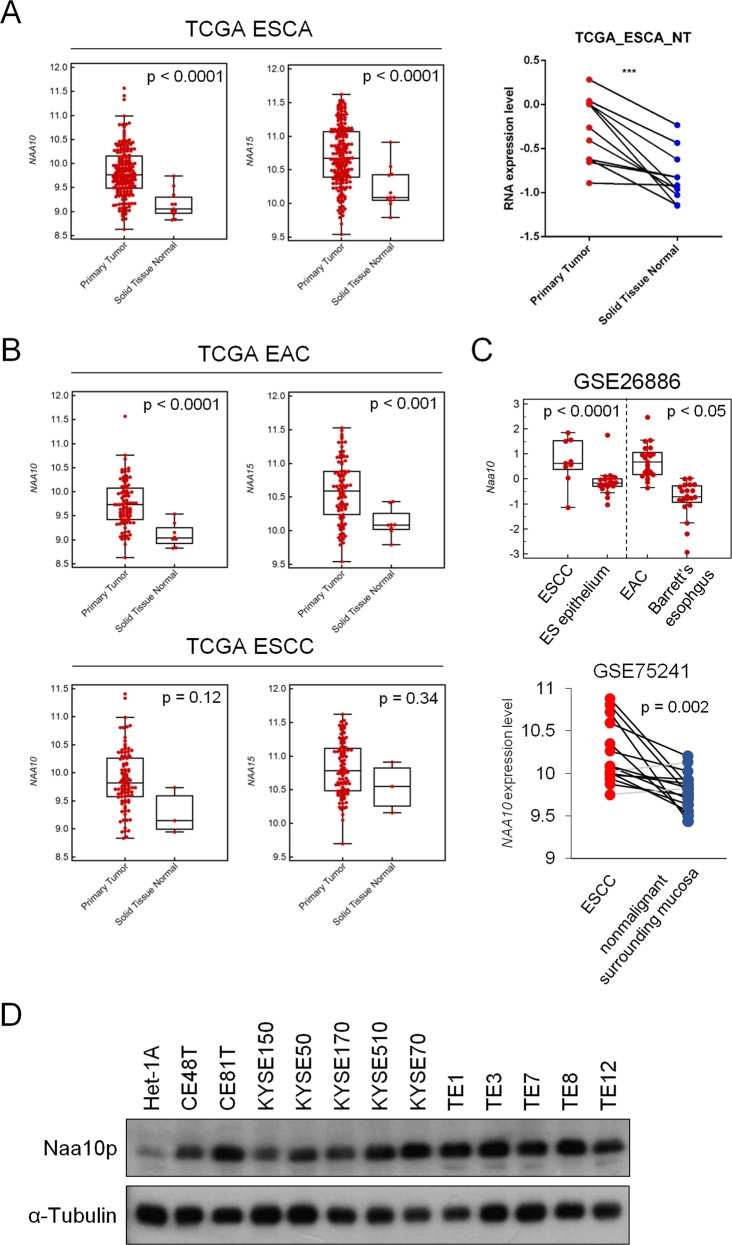
*NAA10* is highly expressed in both esophageal adenocarcinoma (EAC) and esophageal squamous carcinoma (ESCC). **A** Left panel: Comparison of *NAA10/15* expression levels between ESCA and normal tissues. Right panel: Comparison of *NAA10/15* expression levels in paired ESCA and adjacent normal tissues. **B** Comparison of *NAA10/15* expression levels between cancerous and normal tissues. Upper panel: EAC, lower panel: ESCC. **C** Comparison of *NAA10* expression levels between cancerous, premalignant, and normal tissues. **D** Western blot analysis of Naa10p in ESCA cell lines and immortalized ES epithelial cell line, Het-1A.

We first took advantage of The Cancer Genome Atlas (TCGA) database to analyze *NAA10* expression in ESCA patients. As shown in Fig. 1A, *NAA10* mRNA levels were significantly higher in ESCA tumor tissues compared to normal tissues. Almost all the tumor tissues had a higher *NAA10* mRNA level compared to their individually matched normal adjacent tissues (Fig. 1A, right panel), suggesting that *NAA10* upregulation may be a critical event during ESCA carcinogenesis. To be noted, *NAA15* expression was also higher in ESCA compared to normal tissues. We next evaluated the expression of *NAA10* in different ESCA subtypes. Esophageal squamous cell carcinoma (ESCC) is the major cell type of primary esophageal cancer, accounting for 95% of the disease worldwide. Another cell type of esophageal cancer, esophageal adenocarcinoma (EAC), is commonly diagnosed in the Caucasian population and is closely associated with gastroesophageal reflux disease and Barrett’s esophagus. Due to limited normal tissue specimens enrolled in the TCGA database, *NAA10* and *NAA15* mRNA expression were significantly higher in cancerous tissues than in normal tissues only in the EAC cohort, but not the ESCC cohort (Fig. 1B). To verify this result, we surveyed the Gene Expression Omnibus (GEO) database and compared *NAA10* expression in two independent microarray analyses. As shown in Fig. 1C, *NAA10* mRNA expression was significantly higher in ESCC specimens compared with paired or non-paired normal counterparts. Moreover, higher *NAA10* expression was also confirmed in EAC specimens compared to Barrett’s esophagus, the common precancerous status of the esophagus (Fig. 1C upper panel). As we collected 12 ESCC cell lines to compare their Naa10p expression levels with an SV40 large T antigen immortalized esophageal squamous epithelial cell line, Het-1A, all ESCC cell lines expressed higher levels of Naa10p than Het-1A cells (Fig. 1D). Collectively, our results suggest that *NAA10* overexpression might be a common alteration in ESCA cells, both in ESCC and EAC.

### NAA10 expression in ESCA was regulated at both genomic and epigenomic levels

To evaluate the mechanism of *NAA10* overexpression in ESCA cells, genetic alterations of the *NAA10* gene in the TCGA database through the cBioPortal for Cancer Genomics were examined. As shown in Fig. S1A (upper panel), 4% of the total 182 ESCA patients bore genetically altered *NAA10* genes. Among the eight *NAA10*-altered patients, seven have *NAA10* gene amplification and one has *NAA10* deletion. Moreover, a significantly positive correlation (Rho = 0.307, *p* < 0.0001) between the *NAA10* gene copy number and mRNA expression was observed in the TCGA ESCA cohort (Fig. S1A, lower panel). These results suggest that in some ESCA patients, their overexpressed *NAA10* may result from gene amplification events during the carcinogenesis process. Moreover, correlations between CpG islands methylation and *NAA10* expression were also observed in ESCA (Table S1), suggesting that epigenetic regulation may participate in controlling *NAA10* expression in ESCA. To further evaluate this possibility, we compared the methylation levels at CpG islands on the *NAA10* gene locus (Fig. S1B) of normal squamous (NS), Barrett’s esophagus (BE), and EAC from a methylation screening dataset, GSE104707. As shown in Fig. S1C, methylation levels at some CpG islands were both significantly lower in BE and EAC (cg00434413, cg01856640, and cg12521678). Some CpG islands have significantly lower methylation levels only in EAC (cg26791257 and cg23175583). These methylation changes at CpG islands of the *NAA10* gene suggest that hypomethylation of the *NAA10* gene may occur during the carcinogenesis process of ESCA, or at least that of EAC. Taken together, elevated *NAA10* expression in ESCA may be derived from both genetic and epigenetic alterations.

### Oncogenic c-Myc transcriptionally regulates NAA10 expression in ESCA

**Fig. 2:**
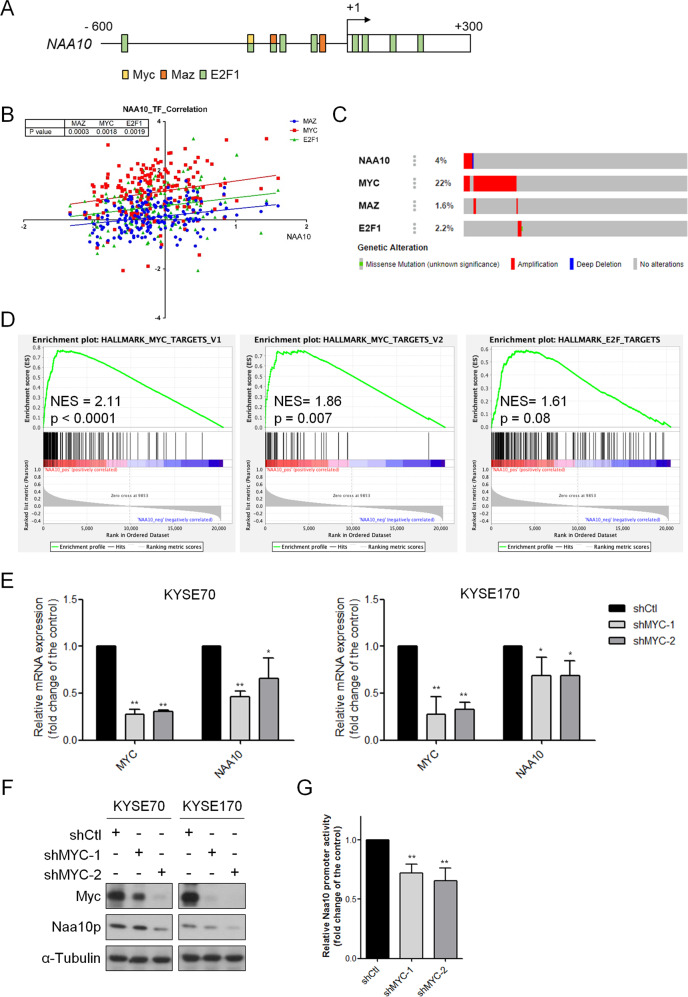
*NAA10* expression in ESCA was regulated by oncogenic c-Myc. **A** Predicted binding motifs of transcription factors on *NAA10* promoter region in UCSC genome browser. **B** Correlation plot comparing RNA levels of transcription factors (*MYC*, *MAZ*, and *E2F1*) and *NAA10* in TCGA. **C** Gene alteration frequencies of *NAA10*, *MYC*, *MAZ*, and *E2F1* in ESCA from TCGA database. **D** GSEA analysis identified the *MYC* signatures among the top ranked activated pathways in *NAA10*-high ESCA patients (TCGA). **E** NAA10 expression in MYC-knockdown KYSE70 and KYSE170 were detected by qPCR. **F** Naa10p expression in c-Myc knockdown KYSE70 and KYSE170 were detected by Western blot. **G** c-Myc-depleted KYSE70 cells and control cells were transfected with *NAA10* reporter and subjected to luciferase activity assays. GSEA gene set enrichment analysis, NES normalized enrichment score.

Since DNA methylation status affected the chromatin condensation and transcriptional activation of genes, we next surveyed transcriptional factors intended to identify critical upstream regulation of *NAA10*. After analyzing the promoter region of *NAA10*, several transcriptional factors were predicted to bind with the *NAA10* promoter and affect its expression in ESCA (Fig. 2A). Positive correlations of *MYC*, *MAZ*, and *E2F1* with *NAA10* expression were observed in the TCGA ESCA database (Fig. 2B). As can be seen in Fig. 2C, *MYC* has the highest genetic alteration ratio (22%) compared to *MAZ* (1.6%) and *E2F1* (2.2%). While the previous finding suggested that nearly 20% of ESCC and 30% of EAC bearing *MYC* gene dysregulation, gene set enrichment analysis (GSEA) of the TCGA ESCA datasets revealed that *MYC* signature is one of the top enriched gene signatures in *NAA10*^Hi^ tumors compared with *NAA10*^Low^ samples (Fig. 2D). In contrast, *MAZ* and *E2F1* signatures did not significantly correlate with *NAA10* expression (Fig. 2D and data not shown). We thus examined the effect of c-Myc depletion in Naa10p expression. Stable knockdown of *MYC* by shRNAs resulted in the reduction of Naa10p expression in both KYSE70 and KYSE170 cells (Fig. 2E, F). To further verify if the *NAA10* promoter activity could be affected by c-Myc, a 1014 bp upstream region (+222 to −792) of the *NAA10* gene was constructed into a luciferase reporter. Figure 2G shows that c-Myc deletion led to a significant reduction of *NAA10* promoter activities. Our results suggest that oncogenic c-Myc may directly regulate Naa10p expression in ESCA.

### High NAA10 levels correlate with poor survival outcomes of ESCA patients

**Fig. 3:**
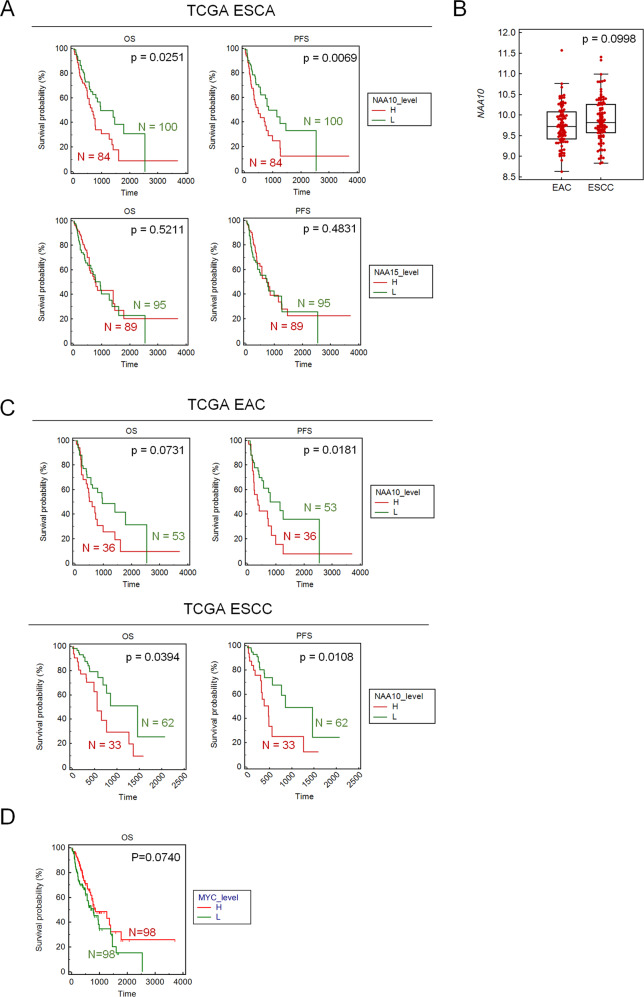
*NAA10* is a prognostic marker of ESCA. **A** Kaplan–Meier plot of overall (OS) and progress-free (PFS) survival of ESCA patients stratified by *NAA10* expression level. A log rank test was used to show differences between groups. **B** Comparison of the *NAA10* expression level between EAC and ESCC. **C** Kaplan–Meier plot of OS and PFS survival of EAC or ESCC patients stratified by *NAA10* expression level. A log rank test was used to show differences between groups. **D** Kaplan–Meier plot of overall (OS) survival of ESCA patients stratified by *MYC* expression level. A log rank test was used to show differences between groups.

To further validate the prognostic values of *NAA10*, the survival probability of patients in the TCGA ESCA database was stratified by *NAA10* expression. As shown in Fig. 3A, patients with higher *NAA10* expression significantly correlated with poorer overall or progress-free survival. In contrast, *NAA15* showed no prognostic values in ESCA patients. Moreover, as we observed slightly higher *NAA10* expression in ESCC compared with that observed in EAC (Fig. 3B), we wondered if the prognostic potentials of *NAA10* in ESCC and EAC were different. The TCGA ESCA patients were separated by their subtypes, and as can be seen in Fig. 3C, both ESCC and EAC patients with higher *NAA10* expression levels had a shorter survival time. Although ESCC and EAC are different in multiple aspects such as cellular origins, histological features, risk factors, and genetic abnormalities, they do share some common features, such as *MYC* alterations [27]. Interestingly, although *MYC* amplification frequently occurs in advanced stages of ESCC, the analysis we did to validate the prognostic value of *MYC* using the TCGA ESCA database showed that *MYC* expression did not correlate with patient survival (Fig. 3D). Therefore, *NAA10*, as a c-Myc-targeted gene may be a more accurate prognostic marker in ESCA.

### Naa10p regulates cell invasive ability of ESCA cells through its acetyltransferase activity

**Fig. 4:**
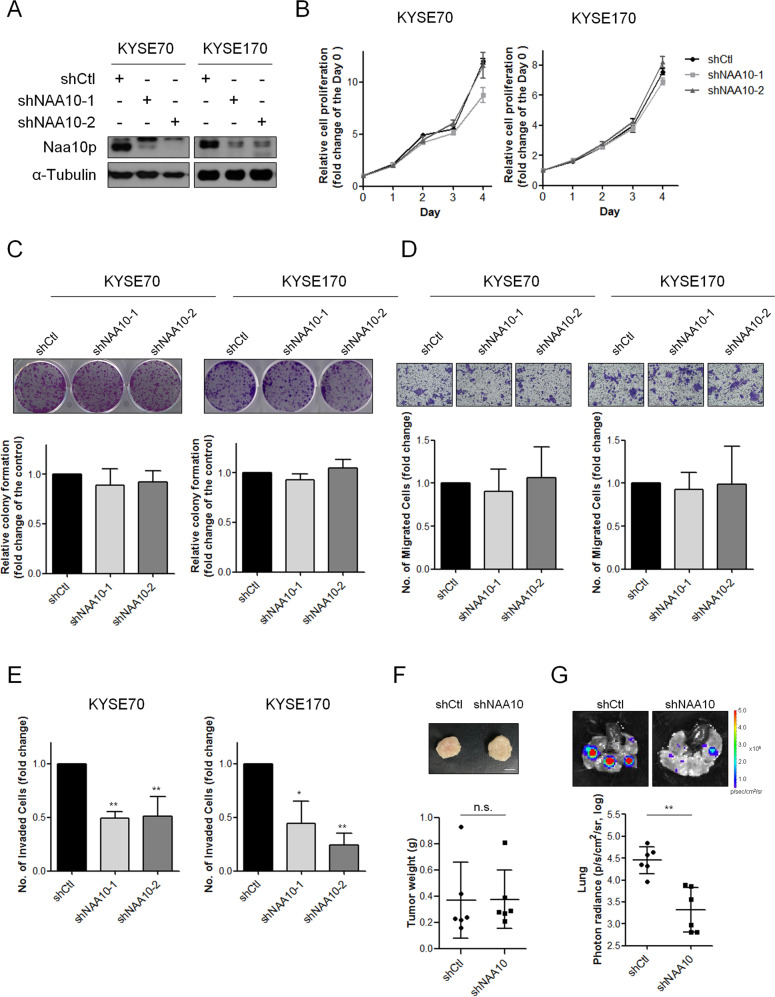
Naa10p regulates the cell invasiveness of ESCA cells. **A** Naa10p protein expression in KYSE70 and KYSE170 cells transfected with either Control-shRNA or *NAA10*-shRNA. **B** Cell proliferation assays of KYSE70 and KYSE170 cells transfected with either Control-shRNA or *NAA10*-shRNA. The data are shown as the relative fold change of the optical density (OD) compared to the baseline OD measured on Day 0. **C** Colony formation assays of KYSE70 and KYSE170 cells transfected with either Control-shRNA or *NAA10*-shRNA. **D** Cell migration assays of KYSE70 and KYSE170 cells transfected with either Control-shRNA or *NAA10*-shRNA. **E** Cell invasion assays of KYSE70 and KYSE170 cells transfected with either Control-shRNA or *NAA10*-shRNA. Differences are shown compared with control cells presented as the mean ± SD of three independent experiments. *P < 0.05, **P < 0.01 when compared to control group by two-tailed Student’s *t* test. **F** KYSE70 cells transfected with either Control-shRNA or *NAA10*-shRNA were subcutaneously injected into NOD/SCIDγ mice. Tumor weight was analyzed. Scale bar: 0.5 cm. Bars are the mean ± SD of indicated (*n*) independent experiments. **G** Representative bioluminescence images of lung metastasis in mice after subcutaneous injection of KTSE70-Luc cells transfected with either Control-shRNA or *NAA10*-shRNA. Scale bar: 0.5 cm. Quantification of lung bioluminescence in photons/second.

Our clinical database analysis supported an oncogenic role of *NAA10* in ESCA; we next examined how Naa10p functions in ESCA cells. The stable knockdown of Naa10p in two esophageal cancer cell lines, KYSE70 and KYSE170 (Fig. 4A), was first evaluated. Proliferative determination of Naa10p in ESCA by performing short-term proliferation curves or long-term colony formation did not show significant differences between control and Naa10p-depleted cells (Fig. 4B, C). Nor was the transwell migration ability of ESCA cells affected by Naa10p depletion (Fig. 4D). However, knockdown of Naa10p significantly reduced the cell invasive ability of ESCA cells (Fig. 4E). Similar results were also obtained when we performed an in vivo subcutaneous metastasis mouse model. The tumor weights did not show a significant difference between Naa10p-silencing xenografts compared with the control xenografts (Fig. 4F). In contrast, metastases in the lung were significantly fewer in Naa10p-silencing groups compared to the shCtl group (Fig. 4G). Consistent with the above results, increased invasiveness of Naa10p-overexpressing KYSE50 and KYSE510 cells was observed (Fig. S2A–B). Taken together, these data indicate a specific regulating role of Naa10p on ESCA cell invasiveness.

**Fig. 5:**
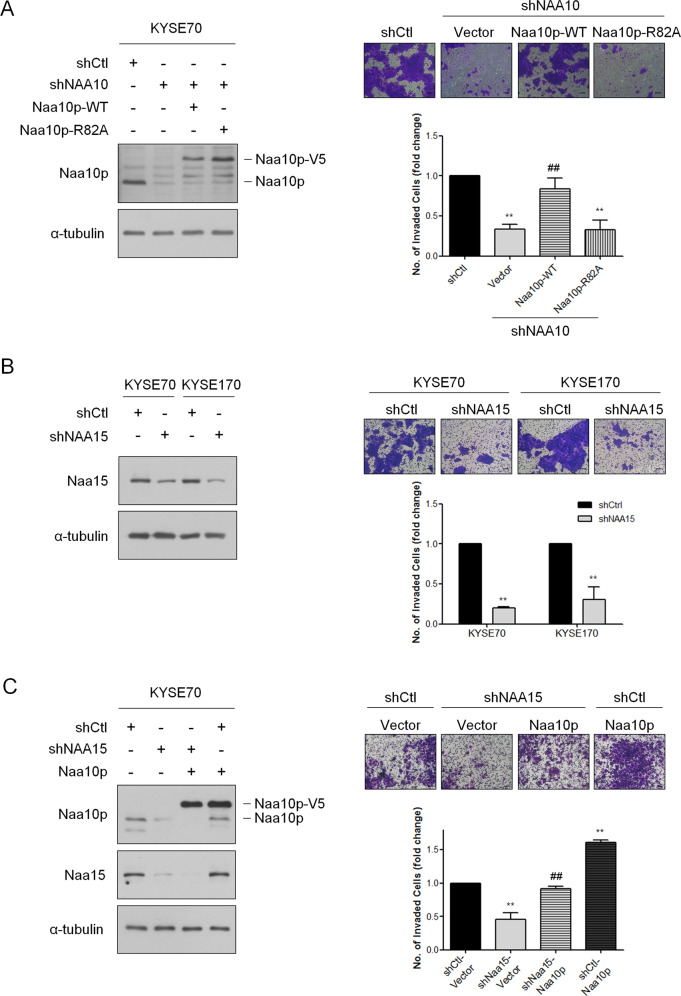
Naa10p regulates ESCA cell invasion via its acetyltransferase activity. **A** Left, KYSE70 cells with stable Naa10p knockdown were transfected with either Naa10p-V5 or Naa10p-R82A-V5 and immunoblotting analysis was performed. Right, cell invasion assays were performed on KYSE70 cells with different levels of Naa10p expression. Bars are mean ± SD of three independent experiments. **P < 0.01 when compared to shCtl group and ## *P* < 0.01 when compared with sh*NAA10*-vector cells by two-tailed Student’s *t* test. Scale bar: 100 μm. **B** Left, the knockdown efficiency of KYSE70 and KYSE170 cells receiving sh*NAA15* or control shRNA. Right, cell invasion assays of KYSE70 and KYSE170 cells transfected with either Ctl-shRNA or *NAA15*-shRNA. The data are shown as relative fold change of invasive cells compared with the shCtl group. **P < 0.01 when compared to the control group by two-tailed Student’s *t* test. Scale bar: 100 μm. **C** Left, KYSE70 cells with stable Naa15 knockdown were transfected with either Vector or Naa10p and immunoblotting analysis was performed. Right, cell invasion assays were performed on stable NAA15-silencing KYSE70 cells transfected with either Vector or Naa10p. The data are shown as relative fold change of invasive cells compared with the shCtl-Vector group. **P < 0.01 when compared to the shCtl-Vector group and ## *P* < 0.01 when compared with sh*NAA15*-Vector cells by two-tailed Student’s *t* test. Scale bar: 100 μm.

Naa10p exerts its N-terminal acetyltransferase activity through cooperation with Naa15p. Naa10p also exists as a monomer that independently acts as a lysine acetyltransferase of diverse target proteins [28]. To confirm whether the acetyltransferase activity of Naa10p is involved in regulating ESCA cell invasive ability, we performed rescuing cell invasion assays by re-expressing Naa10p-V5 or an acetyl-CoA-binding-deficient mutant, R82A-V5, into KYSE70 cells with Naa10p depletion. Naa10p-R82A-V5 lost its ability to associate with acetyl-CoA and exhibited lower acetyltransferase activity. As shown in Fig. 5A, the rescue of Naa10p expression, but not R82A-mutant, significantly increased cell invasive ability in stable Naa10p knockdown ESCA cells, suggesting a unique role of Naa10p acetyltransferase activity in regulating cell invasion of ESCA cells. Since this regulation may depend on either the N-terminal or the lysine acetyltransferase activity of Naa10p, the anchor subunit of the NatA complex, Naa15p, was evaluated. Stable knockdown of Naa15p in both KYSE70 and KYSE170 cells led to a significant decrease in the ability of ESCA cells to invade through an extracellular matrix coating (Fig. 5B). Furthermore, the overexpression of Naa10p-V5 could only reverse the cell invasiveness reduced by Naa15p knockdown to a comparable control group level in KYSE70 cells (Fig. 5C). These results suggested that Naa10p may need to cooperate with Naa15p to regulate cell invasion through acetylating downstream target proteins at the N-terminal amino group in ESCA cells.

### The MYC-NAA10 axis may result in the upregulation of cell invasiveness in ESCA cells

**Fig. 6:**
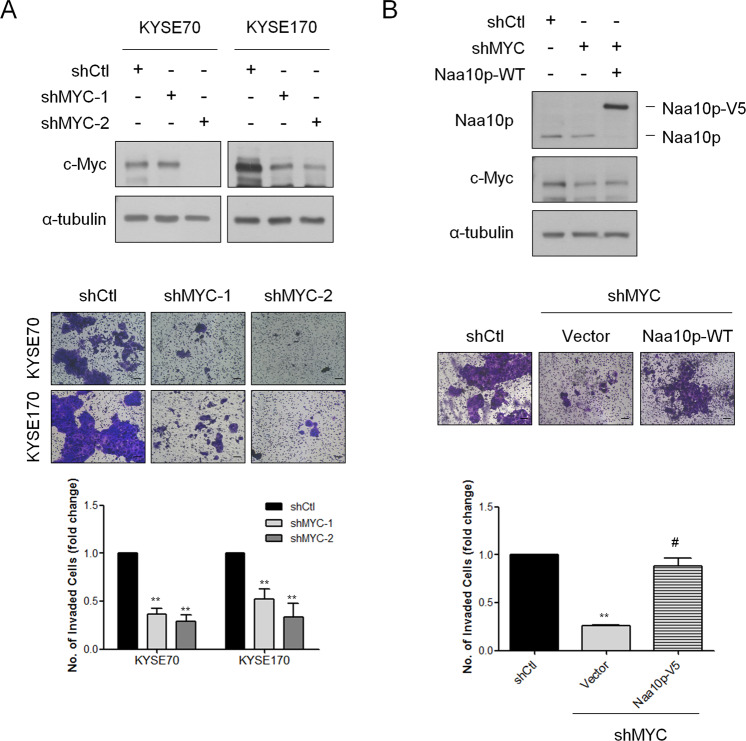
Naa10p mediates c-Myc-induced cell invasiveness. **A** Upper, suppression of c-Myc by shRNAs in KYSE70 and KYSE170 cells were confirmed by immunoblotting analysis. Middle, representative views of cells in the transwell invasion assay. Lower, quantification of cells that invaded through a Matrigel-coated membrane following transfection of Ctl-shRNA or *MYC*-shRNAs. Bars are the mean ± SD of three independent experiments. **P < 0.01 when compared to shCtl group by two-tailed Student’s *t* test. Scale bar: 100 μm. **B** Upper, immunoblotting analysis of stably c-Myc-silencing KYSE70 cells transfected with either vector or Naa10p-V5. Lower, representative images and quantitative data comparing cell invasion of stably c-Myc-silencing KYSE70 cells transfected with either vector or Naa10p-V5. Bars are the mean ± SD of three independent experiments. **P < 0.01 when compared to shCtl group and # *P* < 0.01 when compared with sh*MYC*-vector cells by two-tailed Student’s *t* test. Scale bar: 100 μm.

*MYC* is frequently altered in ESCA and has previously been reported to participate in regulating cell invasiveness of ESCA cells [29]. As our data suggested that *NAA10* may be represented as a novel downstream effector of c-Myc, we next determined whether the *MYC-NAA10* regulatory axis supports invasiveness in ESCA. Transwell invasion assays were first conducted in c-Myc-knockdown KYSE70 and KYSE170 cells. As shown in Fig. 6A, the loss of c-Myc led to a significant reduction in cell invasiveness in both KYSE70 and KYSE170 cells. Furthermore, overexpressing Naa10p did significantly rescue the cell invasive ability reduced by c-Myc-depletion (Fig. 6B). These results confirm that *NAA10* is an important target gene of oncogenic c-Myc signaling and the activation of this *MYC*-*NAA10* axis may result in the elevation of cell invasive ability in ESCA cells.

### Naa10p associates with PAI1 to induce cell invasive ability of ESCA cells

**Fig. 7:**
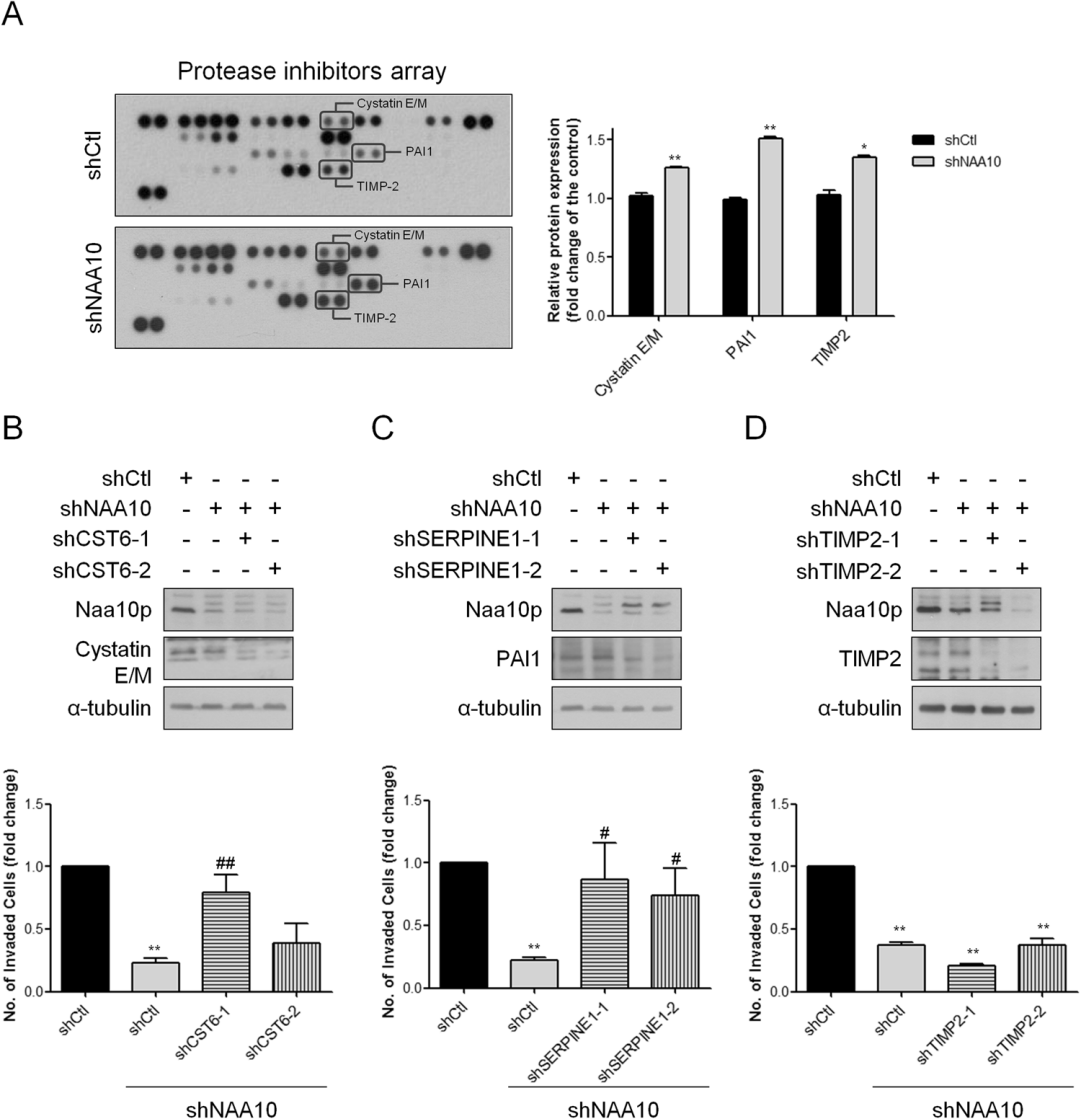
Naa10p regulates cell invasiveness by inducing protease inhibitor expression. **A** Protease inhibitor arrays were used to analyze the expression of protease inhibitors in the cell supernatant of KSYE70 transfected with shCtl or sh*NAA10*. **B** Immunoblotting analysis of stably Naa10p-silencing KYSE70 cells transfected with either Ctl-shRNA or *CST6* shRNAs. Quantitative data comparing cell invasion of stably Naa10p-silencing KYSE70 cells transfected with either Ctl-shRNA or *CST6* shRNAs. Bars are the mean ± SD of three independent experiments. **P < 0.01 when compared to shCtl group and # *P* < 0.05 when compared with sh*NAA10*-shCtl cells by two-tailed Student’s *t* test. **C** Immunoblotting analysis of stably Naa10p-silencing KYSE70 cells transfected with either Ctl-shRNA or *SERPINE1* shRNAs. Quantitative data comparing cell invasion of stably Naa10p-silencing KYSE70 cells transfected with either Ctl-shRNA or *SERPINE1* shRNAs. Bars are the mean ± SD of three independent experiments. **P < 0.01 when compared to shCtl group and ## *P* < 0.01 when compared with sh*NAA10*-shCtl cells by two-tailed Student’s *t* test. **D** Immunoblotting analysis of stably Naa10p-silencing KYSE70 cells transfected with either Ctl-shRNA or *TIMP2* shRNAs. Quantitative data comparing cell invasion of stably Naa10p-silencing KYSE70 cells transfected with either Ctl-shRNA or *TIMP2* shRNAs. Bars are the mean ± SD of three independent experiments. **P < 0.01 when compared to shCtl group and ## *P* < 0.01 when compared with sh*NAA10*-shCtl cells by two-tailed Student’s *t* test.

Since Naa10p specifically affected cell invasive ability of ESCA cells, we next evaluated the expression of proteases and protease inhibitors, which have well-established roles in cell invasion, to reveal the potential downstream mechanism. Proteases and protease inhibitors in Naa10p-manipulated ESCA cells were analyzed by using protein arrays. Three protease inhibitors, including Cystatin E/M, PAI1, and TIMP2, were upregulated in the conditional media from Naa10p-depleted KYSE70 cells (Fig. 7A), implying that these protease inhibitors may participate in Naa10p-regulated cell invasion in ESCA cells. In contrast, although some proteases were found to increase after Naa10p depletion, no decreased proteases were identified (Fig. S3A). To elucidate whether Naa10p regulates the above-identified protease inhibitors and further induces cell invasion, we first analyzed the rescue efficacies in cell invasiveness obtained by introducing these protease inhibitors shRNAs into Naa10p-depleted KYSE70 cells. As shown in Fig. 7B–D, successful reduction of either Cystatin E/M, PAI1, or TIMP2 by shRNAs in Naa10p-depleted KYSE70 cells was confirmed by western blotting. Despite that both PAI1- and Cystatin E/M- silencing show some rescue effects, only the PAI1 knockdown groups showed consistent and significant results in rescuing the cell invasiveness of the Naa10p-depleted cells (Fig. 7C). Since the role of PAI1 in regulating ESCA invasion remains controversial [30], we also tested the effect of PAI1 in our cell model to clarify its role in the cell invasion process. As shown in Fig. S3B, the loss of PAI1 led to significantly increased invasiveness in the KYSE70 cells. The result strengthens the hypothesis that Naa10p may regulate PAI1 expression and further enhance cell invasion of ESCA cells.

**Fig. 8:**
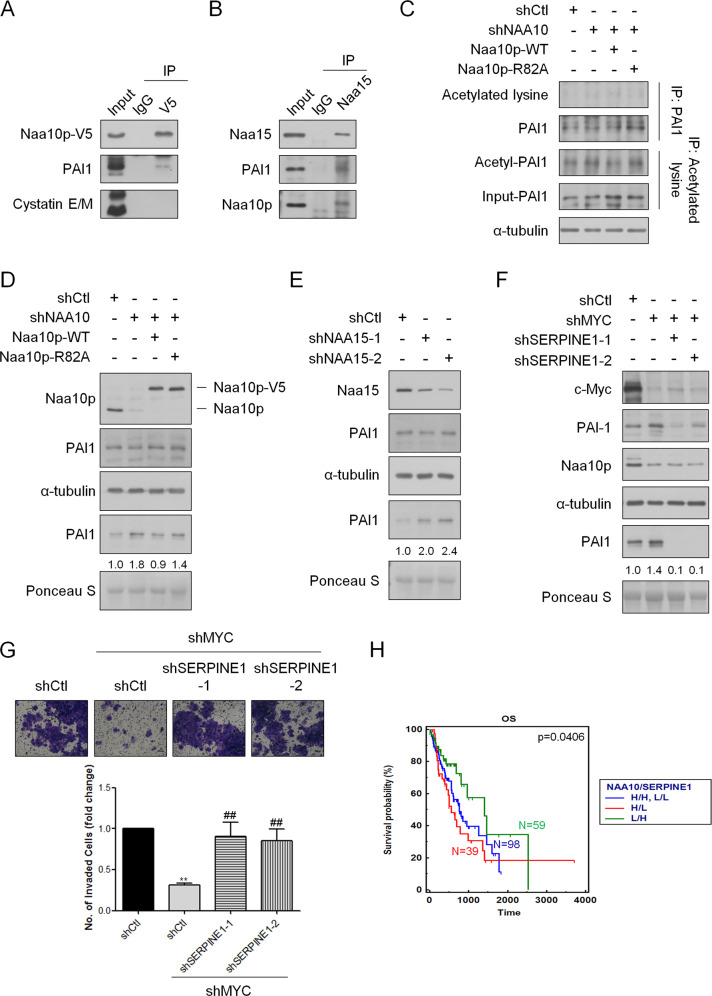
Naa10p associates with PAI1 to regulate cell invasiveness of ESCA cells. **A** Stably Naa10p-V5-expressing KYSE70 cells were immunoprecipitated with an V5 antibody and blotted with anti-PAI1 and anti-Cystatin E/M antibody. **B** KYSE70 cells were immunoprecipitated with Naa15 antibody and blotted with anti-Naa10p and anti-PAI1 antibody. **C** KYSE70 cells with stable Naa10p knockdown were transfected with either Naa10p-V5 or Naa10p-R82A-V5. Cell extracts were immunoprecipitated with an anti-PAI1 antibody or anti-acetylated lysine antibody and blotted with anti-acetylated lysine and anti-PAI1 antibody. **D** KYSE70 cells with stable Naa10p knockdown were transfected with either Naa10p-V5 or Naa10p-R82A-V5. Cell extracts and conditioned medium of these cells were collected and immunoblotting analysis was performed. **E** Cell extracts and conditioned medium of KYSE70 cells receiving sh*NAA15* or control shRNA were collected and immunoblotting analysis was performed. **F** Immunoblotting analysis of stably c-Myc-silencing KYSE70 cells transfected with either Ctl-shRNA or *SERPINE1* shRNAs. **G** Quantitative data comparing cell invasion of stably c-Myc-silencing KYSE70 cells transfected with either Ctl-shRNA or *SERPINE1* shRNAs. Bars are the mean ± SD of three independent experiments. **P < 0.01 when compared to shCtl group and ## *P* < 0.01 when compared with sh*MYC*-shCtl cells by two-tailed Student’s *t* test. Scale bar: 100 μm. **H** Kaplan–Meier plot of overall (OS) survival of ESCA patients stratified by *NAA10* and *SERPINE1* expression level. A log rank test was used to show differences between groups.

To clarify the underlying mechanism, we evaluated the interactions of Naa10p with these protease inhibitors. Co-immunoprecipitation was conducted in Naa10p-V5-overexpressing ESCA cells and only PAI1 was detected in the immune complex precipitated by the anti-V5 antibody (Fig. 8A). The association of Naa10p and Cystatin E/M was not observed, suggesting that PAI1 may act as a more direct downstream target for Naa10p during ESCA progression. Moreover, PAI1 was also detected in the immune complex precipitated by the anti-Naa15 antibody (Fig. 8B), supporting that Naa10p, Naa15p, and PAI1 may form a complex. Since Naa10p exerts its acetyltransferase activity at the N-terminus through cooperation with Naa15p and also no detectable alterations in acetyl-lysine signal of PAI1 were observed in Naa10p-manipulated cells (Fig. 8C), we hypothesized that N-terminal acetylation of PAI1 may exist. We further examined the expression of PAI1 in Naa10p-manipulated KYSE70 cells. Although the mRNA expression of PAI1 (*SERPINE1*) was not altered by depletion of *NAA10* expression (Fig. S3C), the expression of secretory PAI1 protein did increase after silencing of either Naa10p or Naa15p (Fig. 8D, E). These results imply that Naa10p/Naa15p may regulate PAI1 expression only at the post-transcriptional level. In addition, re-expressing Naa10p-V5 but not Naa10p-R82A-V5 decreased the secretory level of PAI1 in stable Naa10p knockdown ESCA cells (Fig. 8D), suggesting that the acetyltransferase activity of Naa10p is important for regulating PAI1 expression.

The regulation axis between c-Myc, Naa10p, and PAI1 was confirmed by knocking down PAI1 expression in stable c-Myc-silencing cells. The expression of Naa10p decreased while the PAI1 protein expression increased in the c-Myc-knockdown group. Stable knockdown of PAI1 rescued the cell invasive ability that was decreased by c-Myc depletion (Fig. 8F, G). All of these observations support that Naa10p acts as a mediating regulator between c-Myc and PAI1 to enhance the cell invasive ability of ESCA. Further investigations were conducted to evaluate the prognostic value of *NAA10* expression alone, *SERPINE1* expression alone, and the use of these two biomarkers in combination. The clinical correlation of Kaplan–Meier survival analysis using the TCGA ESCA samples is shown in Fig. 8H. We separated the patients into three groups by comparing their *NAA10* and *SERPINE1* expression. Patients with a combination of high *NAA10* and low *SERPINE1* expression had significantly poorer overall survival compared to the patients with the combination of low *NAA10* and high *SERPINE1*. Together, these results suggested that c-Myc-Naa10p-PAI1 is a critical axis in regulating cell invasiveness and cancer progression of ESCA.

## Discussion

In this study, we demonstrated the critical regulatory network of Naa10p and its association with either the upstream regulator c-Myc or downstream protease inhibitors during ESCA progression. Our study indicates Naa10p acts as an oncoprotein in regulating ESCA metastasis. Higher *NAA10* expression levels are observed in ESCA patients and correlate with poorer survival. By its acetyltransferase activity, Naa10p may regulate the cell invasive ability of ESCA cells. Moreover, Naa10p/Naa15p complex was observed to interact with PAI1 and their association may lead to the maintenance of PAI1 protein in the cells and further contribute to tumor metastasis. The consequences of the c-Myc-Naa10p-PAI1 axis result in the upregulation of cell invasive ability in ESCA cells, supporting that this regulatory network could either provide more accurate biomarkers or potential therapeutic targets for ESCA treatment.

Recently, Wang et al. reported an opposite effect of Naa10p in regulating tumorigenicity and cell invasion of ESCC [31]. Their findings demonstrated that Naa10p inhibited proliferation and suppressed migration and invasion of TE-1 cells, supporting Naa10p as a tumor suppressor and potential biomarker for ESCC. However, Naa10p was identified from only 4 ESCC specimens and verified in <50 ESCC specimens in their study. Previous reports have shown that clinical analyses of *NAA10* based on smaller cohorts may result in outcomes different from those found in analyses based on bigger cohorts, even in studies of the same tumor type [24, 32]. Our clinical analyses of *NAA10* expression were performed using large online databases including the TCGA database and two GEO datasets, which are composed of >250 ESCA specimens in combination. The inconsistent results may also derive from the experimental designs since, in Wang’s study, only an overexpressing strategy was considered and only one cell line was used to demonstrate the functional effects of Naa10p. The cellular function of Naa10p in our cell models was confirmed by several strategies including overexpression, knockdown, and rescue of Naa10p expression in at least two cell lines. Based on these multiple lines of evidence, we suggest that Naa10p may act as a positive regulator rather than a tumor suppressor in ESCA.

c-Myc is frequently dysregulated in human malignancies including ESCA [33]. As a transcription factor, c-Myc controls selective gene expression to promote various cellular functions including proliferation, apoptosis, mobility, and metabolism. We have identified *NAA10* as a c-Myc downstream target gene by evidence that c-Myc binds to the *NAA10* promoter and further regulates Naa10p expression in ESCA cells. Moreover, the loss of c-Myc led to significant reductions in cell invasive abilities while overexpressing Naa10p significantly rescued the cell invasion of c-Myc-depleted ESCA cells, suggesting that this regulation axis may control cell invasiveness of ESCA cells. Although c-Myc has frequently reported to be overexpressed in ESCA and is recognized as a cancer marker, the prognostic significance of c-Myc expression levels in ESCA remains debated [34, 35]. In fact, unlike the significant prognostic potential of *NAA10* in ESCA, the expression of *MYC* did not correlate with patient survival when analyzing the TCGA ESCA database. Given that lymph node metastasis and distant metastasis are among the most important prognostic factors in ESCA [36, 37] and that Naa10p specifically regulates cell invasiveness and tumor metastasis of ESCA, Naa10p may represent a more suitable and potent prognostic marker for ESCA patients. Furthermore, despite being an obvious target, inhibiting c-Myc therapeutically has proved to be challenging, and practicable inhibition of this protein with pharmaceuticals has yet to be achieved [38]. As we have demonstrated here that the pro-metastasis effect of Naa10p comes from its acetyltransferase activity, it may be feasible to develop Naa10p-inhibitory strategies to target the c-Myc signaling axis in ESCA.

Accumulated evidence suggests that Naa10p is a multifunctional protein, which includes both enzymatic and non-enzymatic mechanisms, involved in various cellular behaviors [39, 40]. The positive and negative effects of Naa10p in regulating cell motility and invasiveness of cancer cells are both documented. These contrary actions of Naa10p may mainly depend on which interaction partners it binds. For example, Naa10p interacts with PIX proteins to inhibit Cdc42/Rac1 activity and thus suppress cell motility and tumor metastasis [24]. Naa10p can also bind with IKKα to inhibit EMT via TGF-β1-Smad3 signaling [41]. Myosin light chain kinase (MLCK), which plays a central role in controlling actin-myosin interaction, was found to be acetylated and suppressed by Naa10p and thus resulted in decreased cell migration [42]. In contrast, Naa10p can form a complex with ADAM9 to promote cell invasiveness of androgen-independent prostate cancer [43]. We have recently identified MMP-2 as an N-terminal acetyltransferase substrate of Naa10p in osteosarcoma. N-acetylated MMP-2 becomes stabilized, thereby promoting cell invasiveness and tumor metastasis [26]. In this study, we found that Naa10p and Naa15p form a complex with PAI1, a member of the serine protease inhibitors, to increase the cell invasive ability of ESCA. The secretory levels of PAI1 were highly dependent on the acetyltransferase activity of Naa10p and the integrity of the Naa10p/Naa15p complex, suggesting that PAI1 may be N-terminal acetylated by NatA. Since most secretory proteins are directed to the lumen of the endoplasmic reticulum to remove their signal peptide in the N-terminus [44], N-terminal processing, especially N-terminal acetylation, would be expected to alter the overall positive charge and further block the translocation of secretory proteins [44, 45]. Although further validation is required, our findings suggest the potential of PAI1 as a novel Naa10p and NatA target; its secretion might be abrogated upon acetylation, thus eliminating its capacity to block invasion.

PAI1 has been reported to execute drastically different functions in various cancer types [46]. A well-established function of PAI1 is to block the conversion of plasminogen to plasmin triggered by tissue-type plasminogen activator (tPA) or urokinase-type plasminogen activator (uPA) [47]. Downregulation of tPA or uPA expression by PAI1 was found to inhibit tumor metastasis, which further supports PAI1 as a cancer inhibitor [48]. Moreover, PAI1 was reported to interact with the integrin-binding site of vitronectin and further inhibit cellular migration [49]. Our study revealed that PAI1 may be presented as an inhibitor for ESCA metastasis by evidence that stable knockdown of PAI1 increased the ability of ESCA to invade through the extracellular matrix. Reduction of PAI1 also significantly rescued cell invasiveness depleted by Naa10p knockdown. However, in previous studies of PAI1 in ESCC, it was characterized more as a promoter of tumor progression. Cancer-associated fibroblasts (CAFs) that secreted PAI1 were found to promote cell invasion, tumor growth, and cisplatin-resistance in ESCC [30, 50]. Such paradoxical roles of PAI1 were also observed among other gastrointestinal cancers [51, 52]. Interestingly, accumulated studies suggest that the effect of PAI1 on tumor progression depends on many aspects, including the concentration, the location, and even the presence of integrins [53–55]. Although further comparisons of PAI1 concentration, PAI1 interaction partners, and PAI1 location in different statuses of ESCA patients may offer more information, the evidence we provide here supports that PAI1 is an invasion-suppressive protein in ESCA cells.

Developing more effective treatments to eliminate cellular invasion and cancer metastasis requires a mechanistic understanding of pathways driving cancer cell invasion. Our results suggest that depletion of Naa10p expression or reduction of Naa10p acetyltransferase activity could lead to diminished cancer cell invasion. Our finding also highlights c-Myc, a critical oncoprotein in ESCA, as an important upstream regulator which controls Naa10p expression. Moreover, since we observed that the cell invasiveness of ESCA depends on the association and regulation of the Naa10p/Naa15p complex toward PAI1 protein, destroying this complex by either inhibiting the association between Naa10p and PAI1 or reducing the Naa10p enzyme activity may result in the reduction of cancer cell invasion. In summary, the association of PAI1/Naa10p/Naa15p and the signaling axis of c-Myc/Naa10p/PAI1 suggest that Naa10p has great potential as both a reliable prognostic factor and a novel therapeutic target for ESCA patients.

## Supplementary information

Supplementary figures and tables

Full and uncropped western blots

checklist

## Data Availability

All data supporting the findings of this study are available from the corresponding author upon reasonable request.
