# Supplementary material for: Naa10p promotes cell invasiveness of esophageal cancer by coordinating the c-Myc and PAI1 regulatory axis
Source: Cell Death Dis. 2022 Nov 24;13(11):995. doi: 10.1038/s41419-022-05441-0 (PMC9700753; doi:10.1038/s41419-022-05441-0)
Supplement: Supplementary file 1 — Supplementary figures and tables [file 41419_2022_5441_MOESM1_ESM.docx]

**Supplementary data for**

**Naa10p promotes cell invasiveness of esophageal cancer by coordinating the c-Myc and PAI1 regulatory axis.**

**The PDF file includes**:

Figure S1. *NAA10* expression in ESCA was controlled via alterations in copy number and/or DNA methylations.

Figure S2. Overexpression of Naa10p results in up-regulation of cell invasion of ESCA.

Figure S3. The regulation of Naa10p on PAI1 expression and the effect of PAI1 on cell invasiveness in ESCA.

Table S1. Correlations of DNA methylation level and *NAA10* expression in ESCA.

Table S2. Sequences of qPCR primers.

**
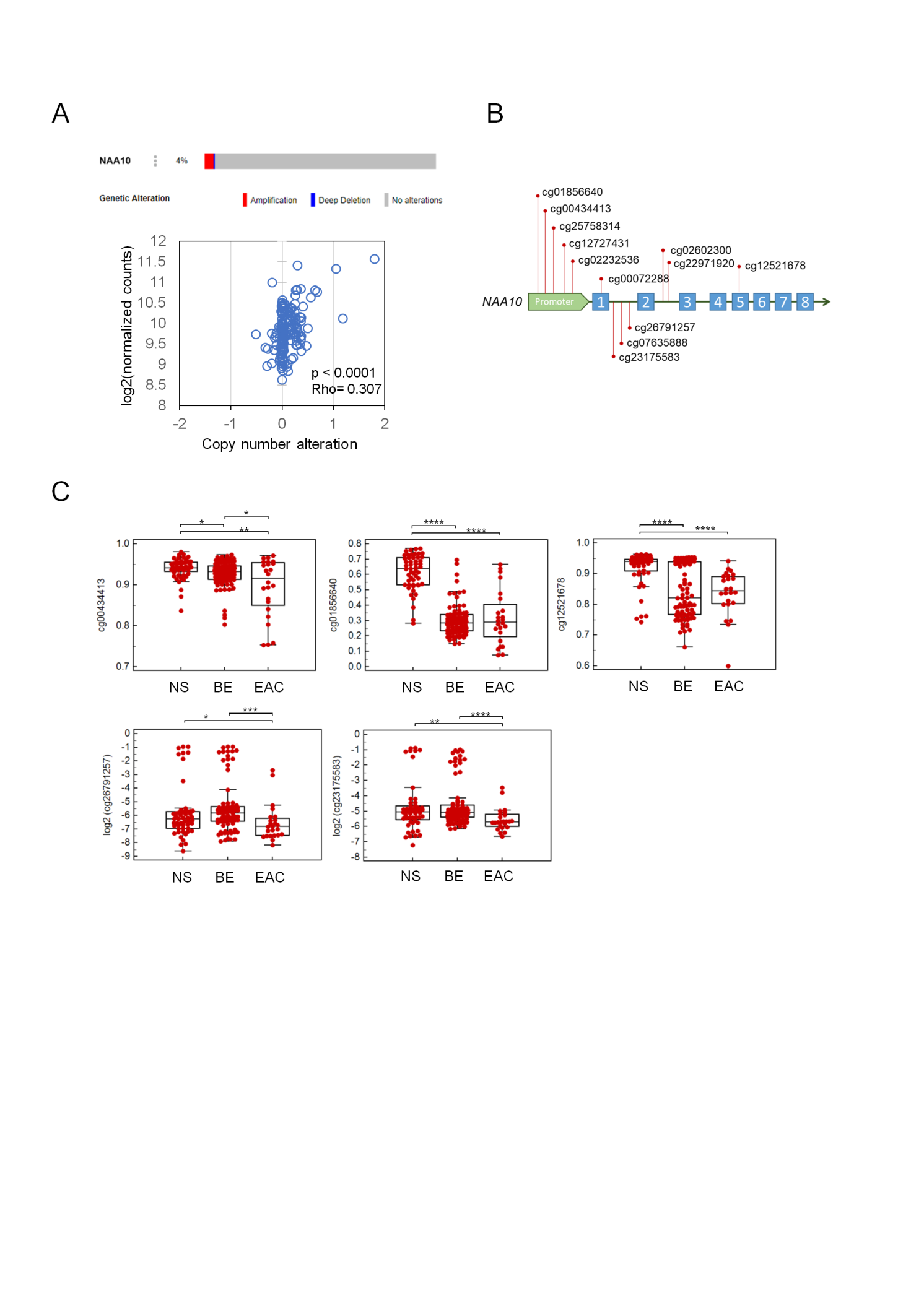
**

**Figure S1. *NAA10* expression in ESCA was controlled via alterations in copy number and/or DNA methylations. (A)** Upper, *NAA10* gene alteration frequency in ESCA from TCGA database. Lower, correlation between copy number and mRNA expression levels of *NAA10* in ESCA. **(B)** Schematic diagram represents the location of CpG islands in the *NAA10* gene locus. **(C)** Comparison of the indicated CpG methylation levels in normal squamous (NS), Barrett’s esophagus (BE), and EAC. Data was acquired from GSE104707. *p < 0.05, **p <0.01, ***p < 0.005 , ****p < 0.0001.

**
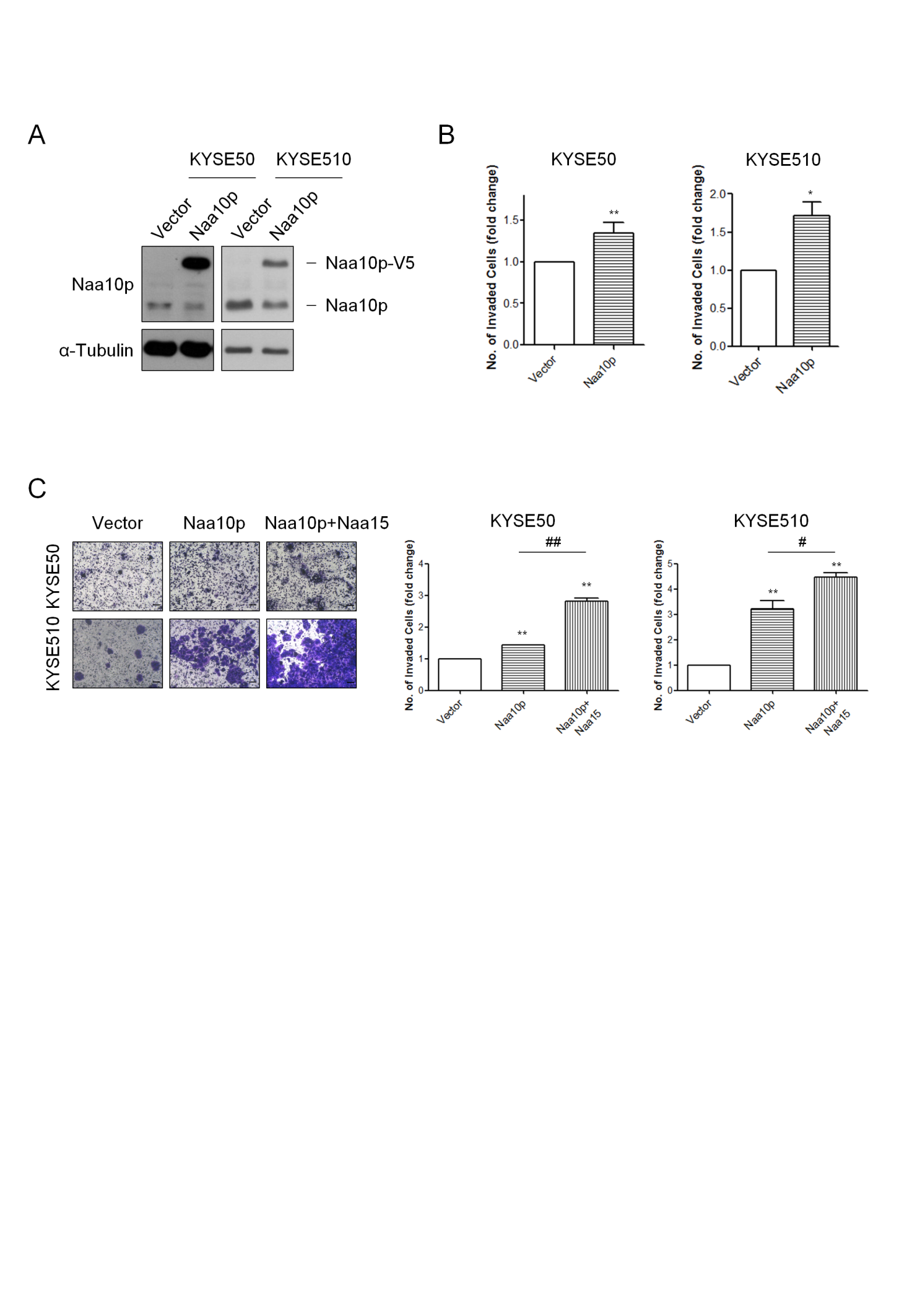
**

**Figure S2. Overexpression of Naa10p results in up-regulation of cell invasion of ESCA (A)** KYSE50 and KYSE510 cells were overexpressed with Naa10p and immunoblotting analysis was performed. **(B)** Cell invasion assay were performed in the KYSE50 and KYSE510 cells with Naa10p overexpression. Bars are mean±SD of three independent experiments. ***P* ＜0.01 when compared to vector group by two-tailed Student’s *t* test.

**
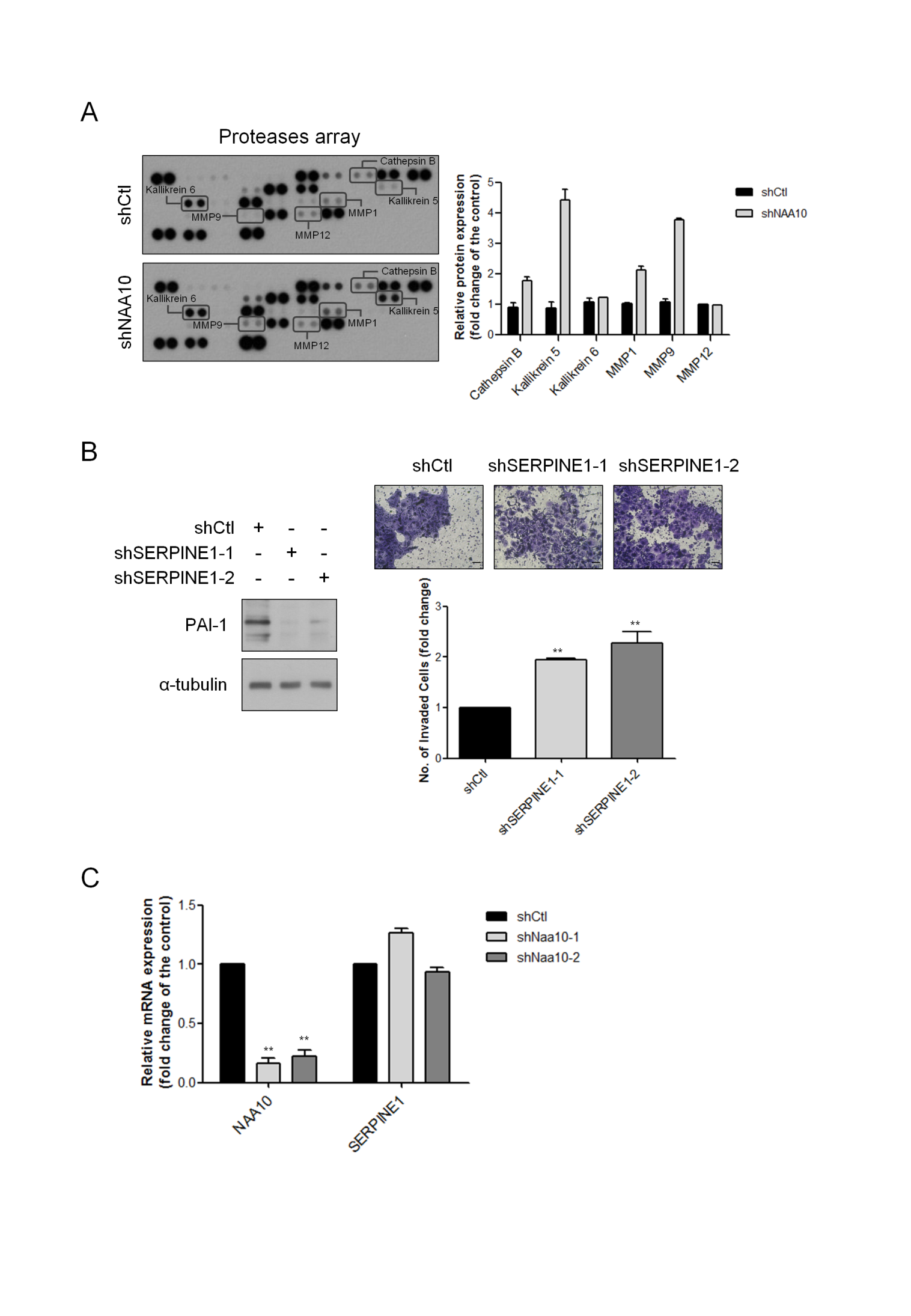
**

**Figure S3. The regulation of Naa10p on PAI1 expression and the effect of PAI1 on cell invasiveness in ESCA.** **(A)** Protease assays were used to analyze the expression of proteases in the cell supernatant of KSYE70 transfected with shCtl or sh*NAA10*. **(B)** Cell invasion assays of KYSE70 cells transfected with either shCtl or sh*SERPINE1*. The data are shown as the relative fold change of invasive cells compared with the shCtl group. ***P* ＜0.01 when compared to shCtl group by two-tailed Student’s *t* test. Scale bar: 100 μm. **(C)** Relative *NAA10* and *SERPINE1* mRNA expression in KYSE70 cells transfected with either shCtl or sh*NAA10*.

Table S1. Correlations of DNA methylation level and *NAA10* expression in ESCA.

| CpG island | Location | *NAA10* correlation | Significance |
| --- | --- | --- | --- |
| cg01856640 | Promoter | negative | ‒ |
| cg00434413 | Promoter | negative | ‒ |
| cg25758314 | Promoter | negative | p < 0.05 |
| cg12727431 | Promoter | negative | p < 0.05 |
| cg02232536 | Promoter | negative | p < 0.05 |
| cg00072288 | Exon 1 | negative | p < 0.05 |
| cg23175583 | Intron 1 | negative | p < 0.05 |
| cg07635888 | Intron 1 | negative | p < 0.05 |
| cg26791257 | Intron 1 | negative | p < 0.05 |
| cg02602300 | Intron 2 | negative | p < 0.01 |
| cg22971920 | Intron 2 | negative | p < 0.01 |
| cg12521678 | Exon 5 | negative | ‒ |

Table S2. Sequences of qPCR primers.

| Gene | Forward primer (5’ to 3’) | Reverse primer (5’ to 3’) |
| --- | --- | --- |
| ***NAA10*** | CAGTGAAGTGGAGCCCAAAT | GAGGTGAATTGCCTTTGCTC |
| ***MYC*** | GGTGCTCCATGAGGAGACA | CCTGCCTCTTTTCCACAGAA |
| ***SERPINE1*** | CTCATCAGCCACTGGAAAGGCA | GACTCGTGAAGTCAGCCTGAAAC |
| ***ACTIN*** | GGCGGCACCACCATGTACCCT | AGGGGCCGGACTCGTCATACT |
