# Supplementary material for: Naa10p promotes cell invasiveness of esophageal cancer by coordinating the c-Myc and PAI1 regulatory axis
Source: Cell Death Dis. 2022 Nov 24;13(11):995. doi: 10.1038/s41419-022-05441-0 (PMC9700753; doi:10.1038/s41419-022-05441-0)

Figure 1.

D

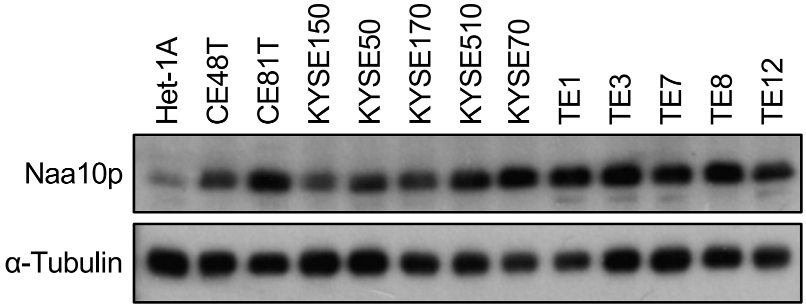

Het-1A
CE48T
CE81T
KYSE150
KYSE50
KYSE170
KYSE510
KYSE70
TE1
TE3
TE7
TE8
TE12
Naa10p
α-Tubulin

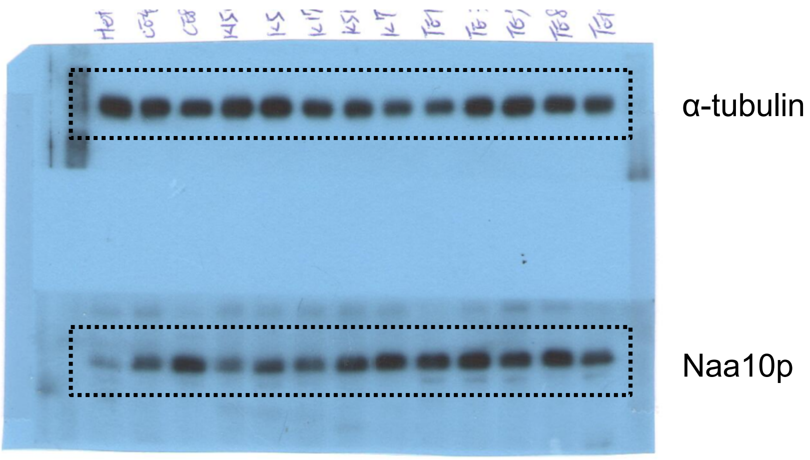

α-tubulin
Naa10p

Figure 2.

F

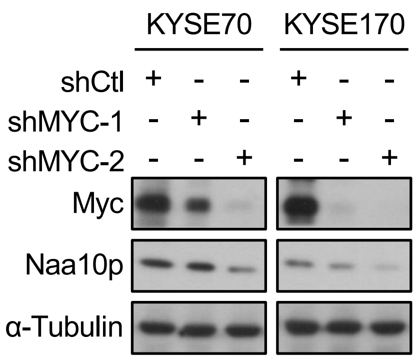
KYSE70
KYSE170
shCtl
shMYC-1
shMYC-2
Myc
Naa10p
α-Tubulin

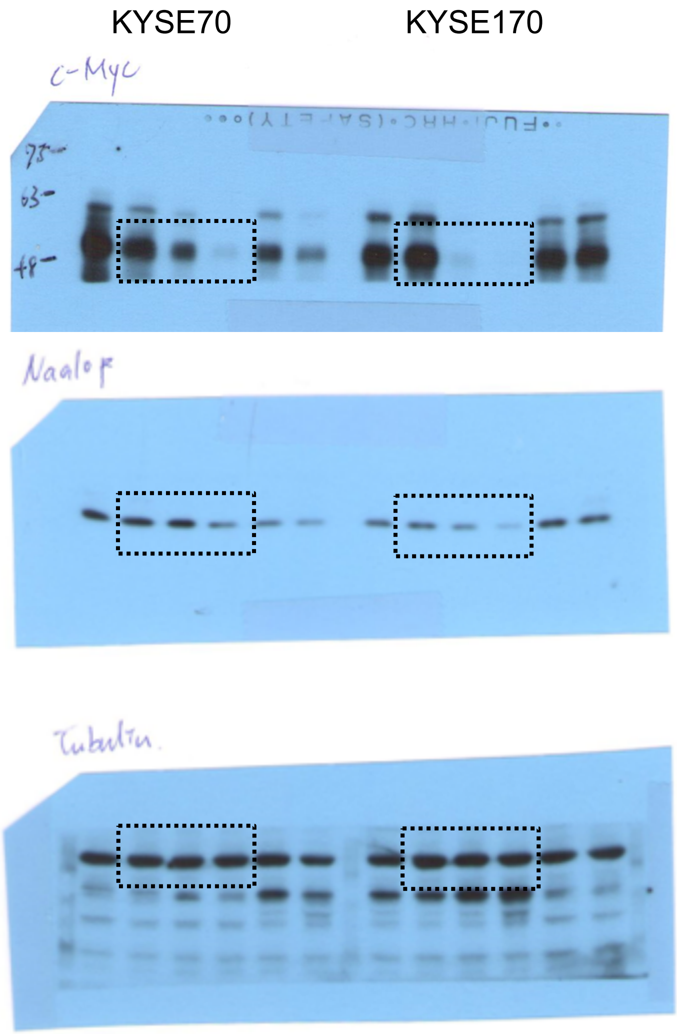
KYSE70
KYSE170
c-Myc
75
63
48
Tubulin

Figure 4.

A

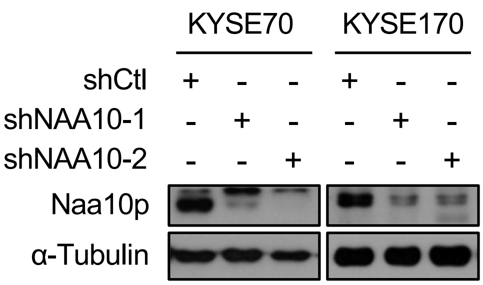

KYSE70
KYSE170
shCtl + - - + - -
shNAA10-1 - + - - + -
shNAA10-2 - - + - - +
Naa10p
α-Tubulin

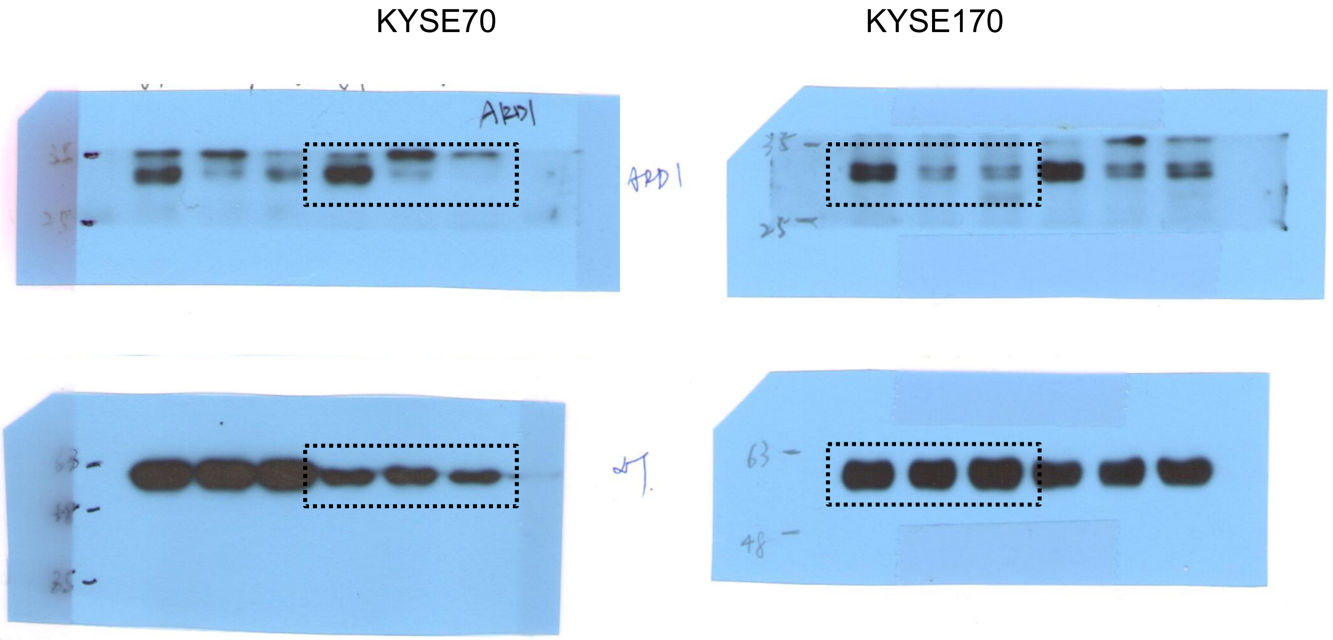

KYSE70
KYSE170

Figure 5.

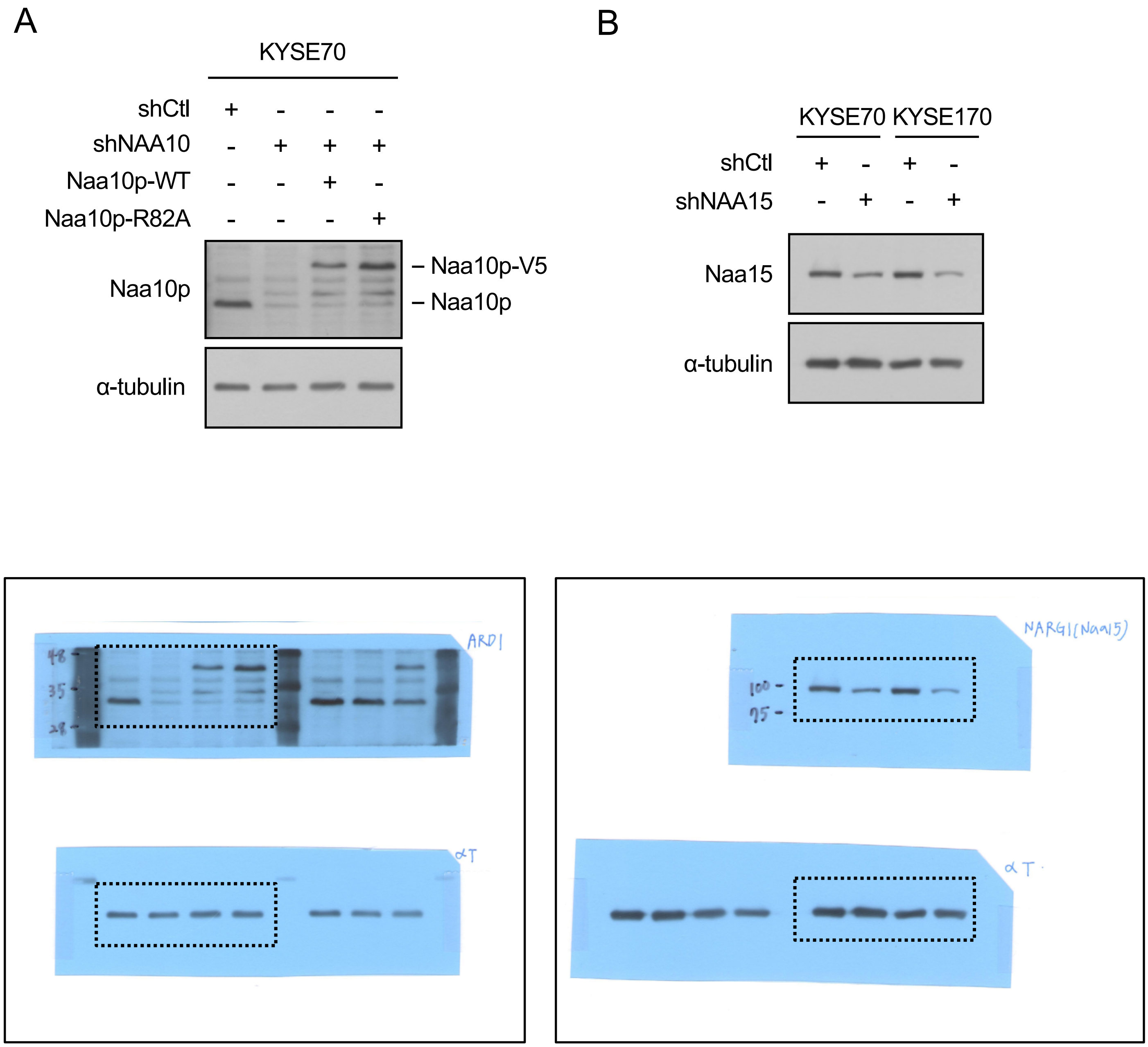

A
KYSE70
shCtl + - - -
shNAA10 - + + +
Naa10p-WT - - + -
Naa10p-R82A - - - +
Naa10p
– Naa10p-V5
– Naa10p
α-tubulin
B
KYSE70 KYSE170
shCtl + - + -
shNAA15 - + - +
Naa15
α-tubulin
ARD1
48
35
28
αT
NARG1(Naa15)
100
75
αT

Figure 5.

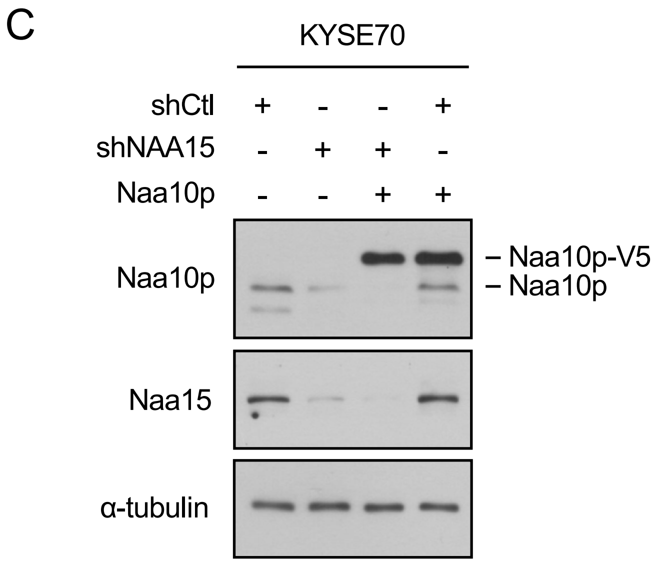
C
KYSE70
shCtl + - - +
shNAA15 - + + -
Naa10p - - + +
Naa10p
Naa15
α-tubulin
Naa10p-V5
Naa10p

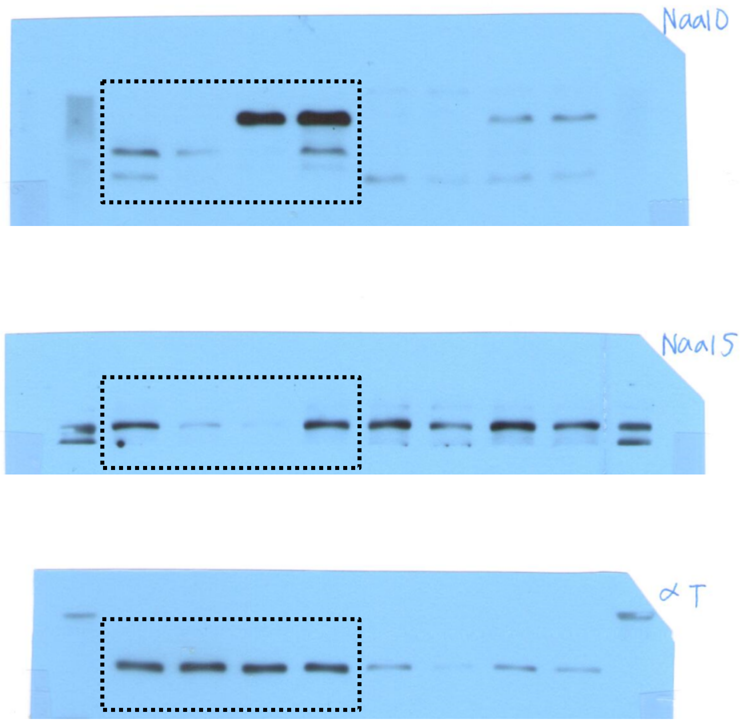
Naa10
Naa15
α T

Figure 6.

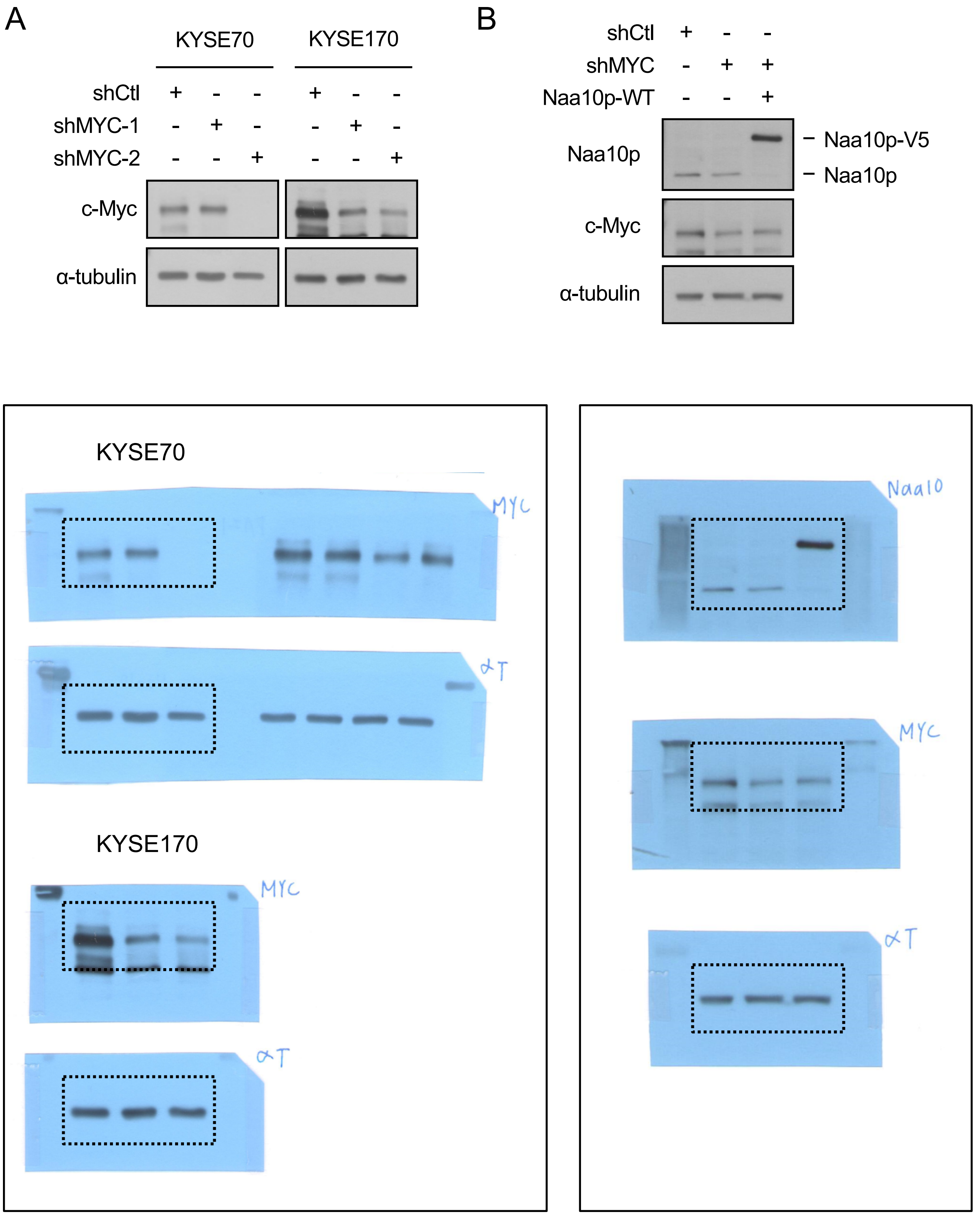
A
KYSE70
KYSE170
shCtl + - - + - -
shMYC-1 - + - - + -
shMYC-2 - - + - - +
c-Myc
α-tubulin
B
shCtl + - -
shMYC - + +
Naa10p-WT - - +
Naa10p
Naa10p-V5
Naa10p
c-Myc
α-tubulin
KYSE70
MYC
αT
KYSE170
MYC
αT
Naa10
MYC
αT

Figure 7.

Figure S3.

A

Protease inhibitors array

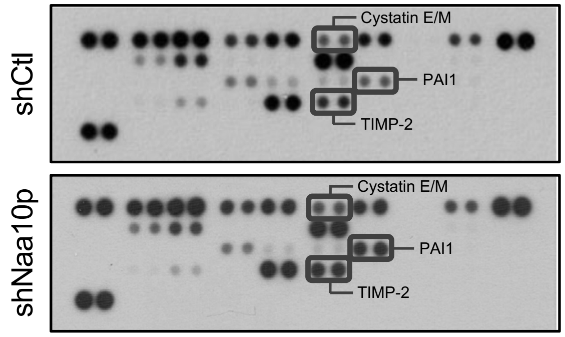

A

Proteases array

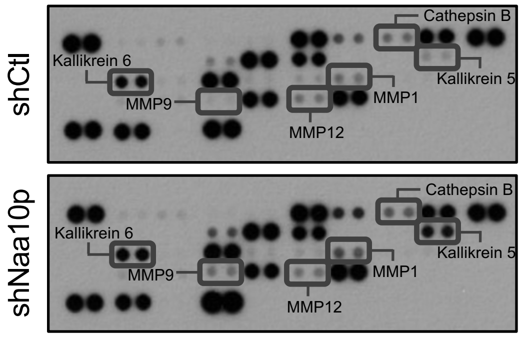

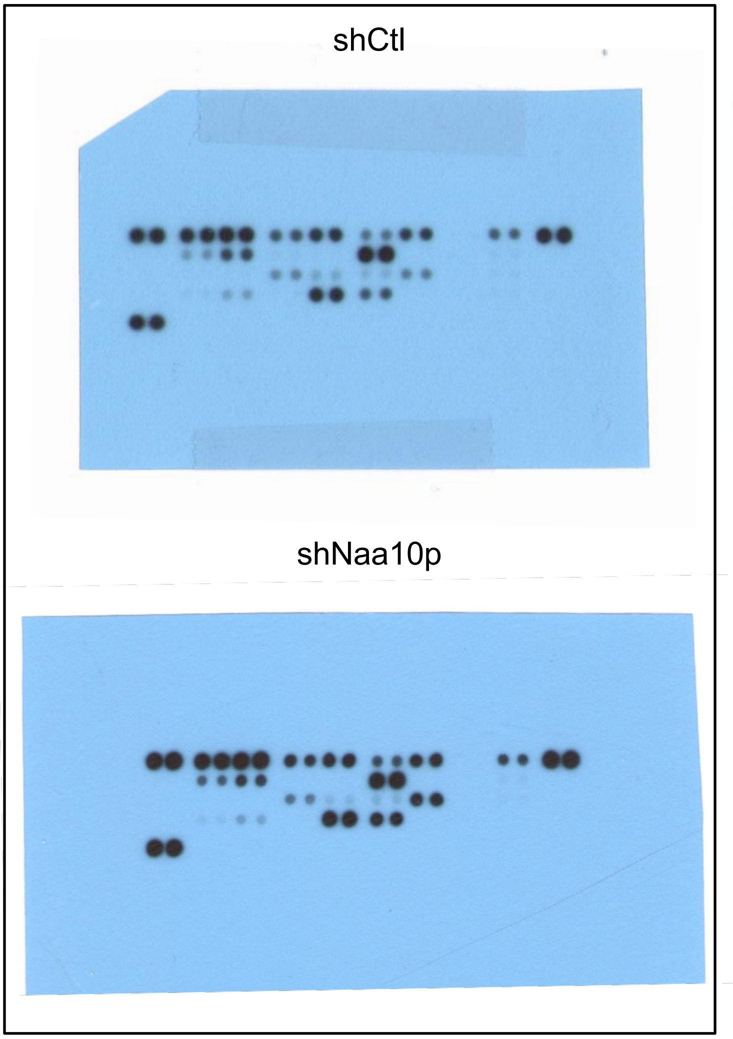

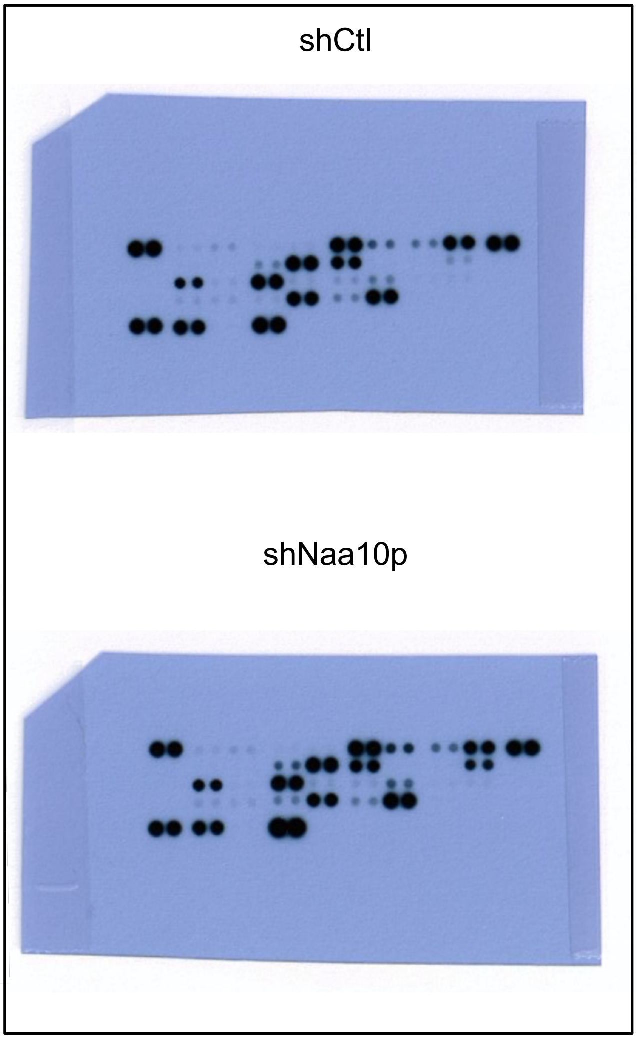

Figure 7.

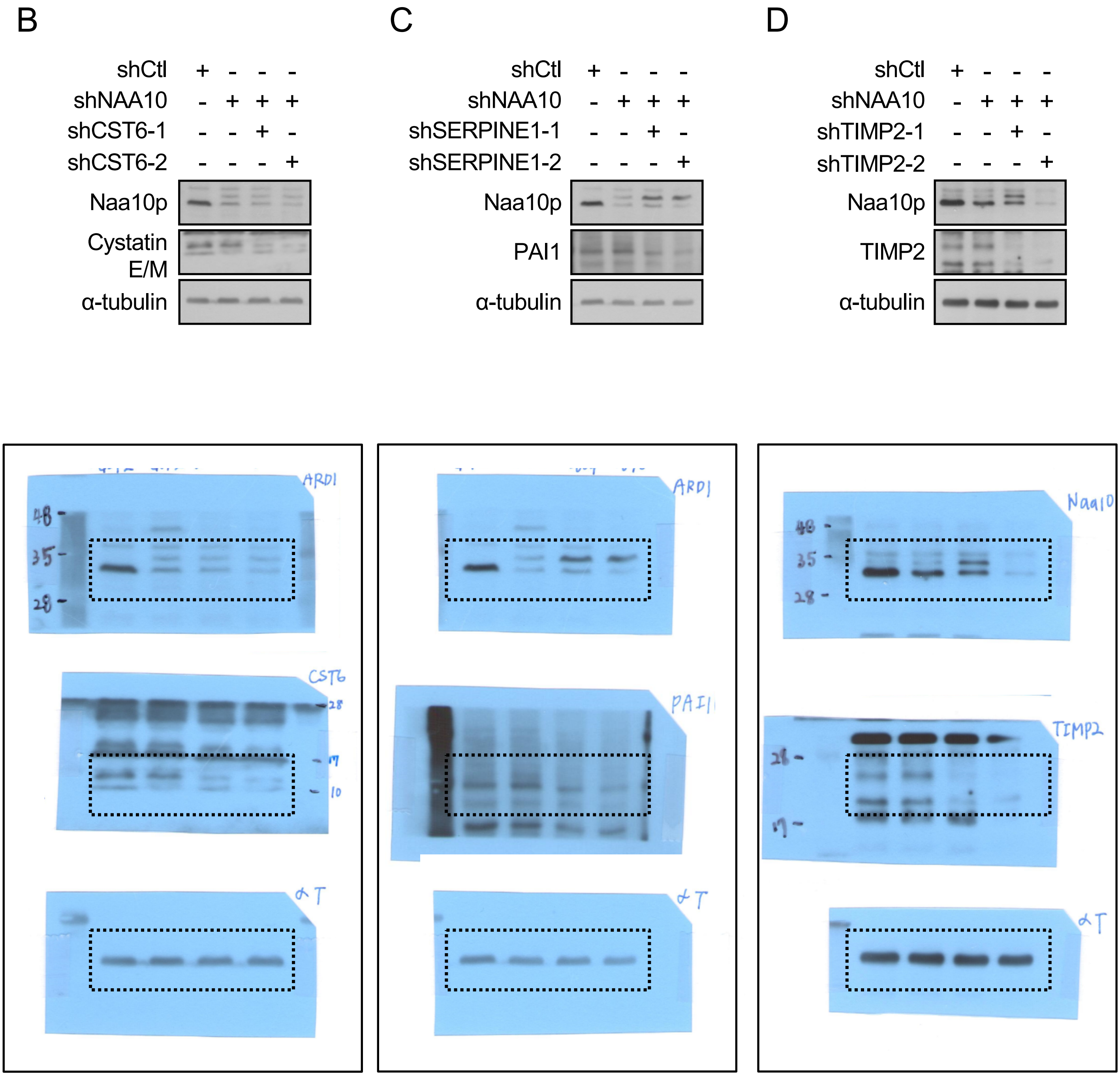

B
shCtl + - - -
shNAA10 - + + +
shCST6-1 - - + -
shCST6-2 - - - +
Naa10p
Cystatin E/M
α-tubulin
C
shCtl + - - -
shNAA10 - + + +
shSERPINE1-1 - - + -
shSERPINE1-2 - - - +
Naa10p
PAI1
α-tubulin
D
shCtl + - - -
shNAA10 - + + +
shTIMP2-1 - - + -
shTIMP2-2 - - - +
Naa10p
TIMP2
α-tubulin

Figure 8.

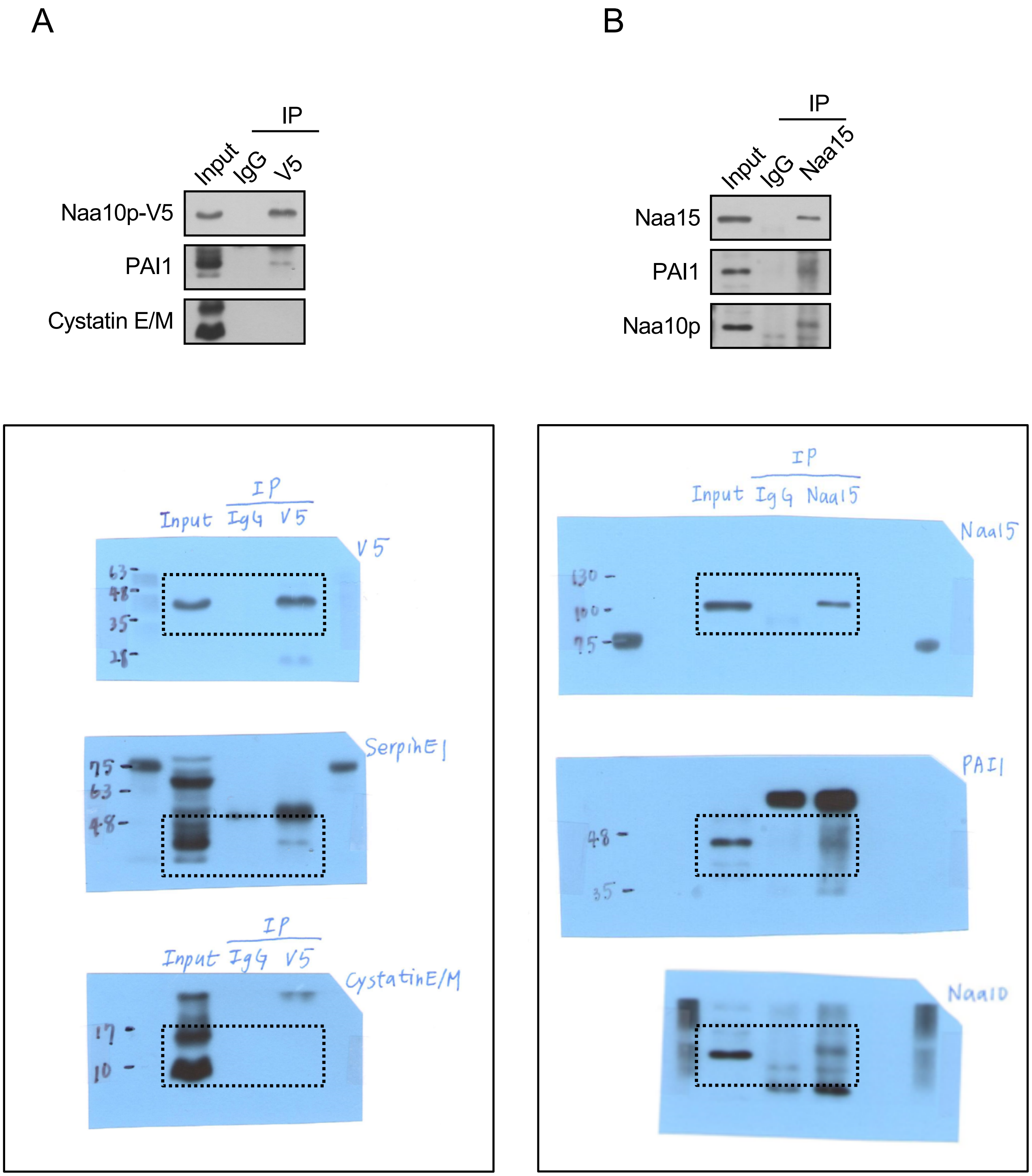
A
IP
Input
IgG
V5
Naa10p-V5
PAI1
Cystatin E/M
B
IP
Input
IgG
Naa15
Naa15
PAI1
Naa10p

Figure 8.

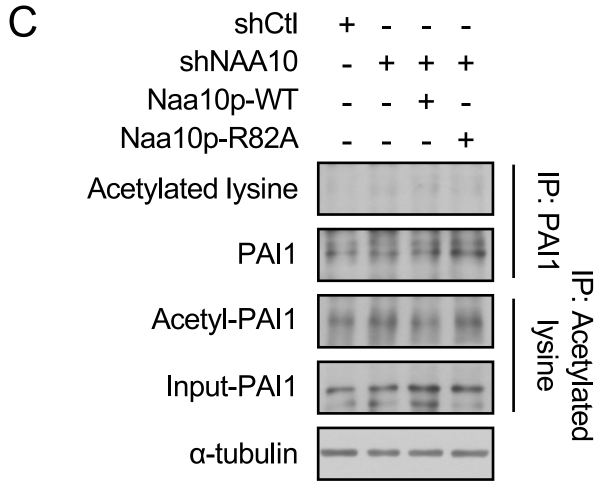
C
shCtl + - - -
shNAA10 - + + +
Naa10p-WT - - + -
Naa10p-R82A - - - +
Acetylated lysine
PAI1
Acetyl-PAI1
Input-PAI1
α-tubulin
IP: PAI1
IP: Acetylated lysine

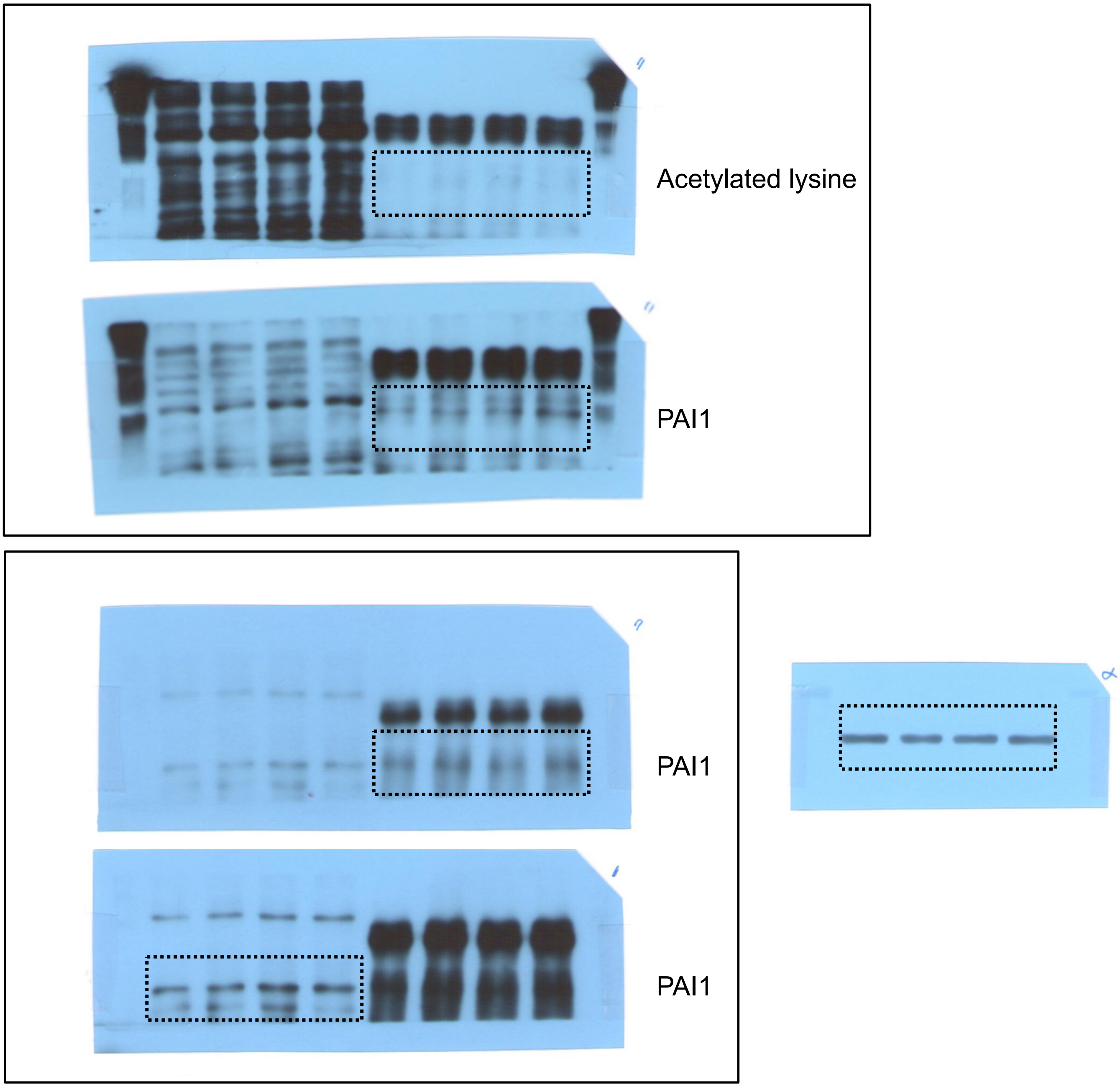
Acetylated lysine
PAI1
PAI1
PAI1
αT

Figure 8.

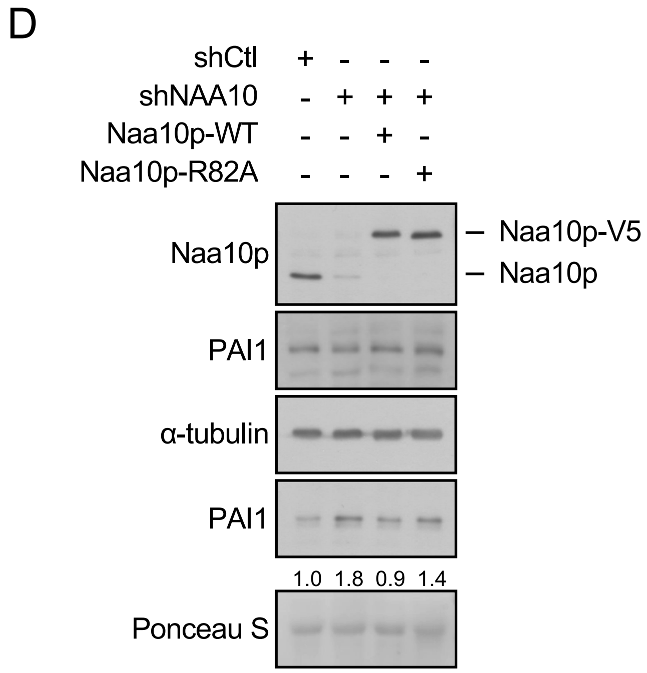
D
shCtl + - - -
shNAA10 - + + +
Naa10p-WT - - + -
Naa10p-R82A - - - +
Naa10p
Naa10p-V5
Naa10p
PAI1
α-tubulin
PAI1
1.0 1.8 0.9 1.4
Ponceau S

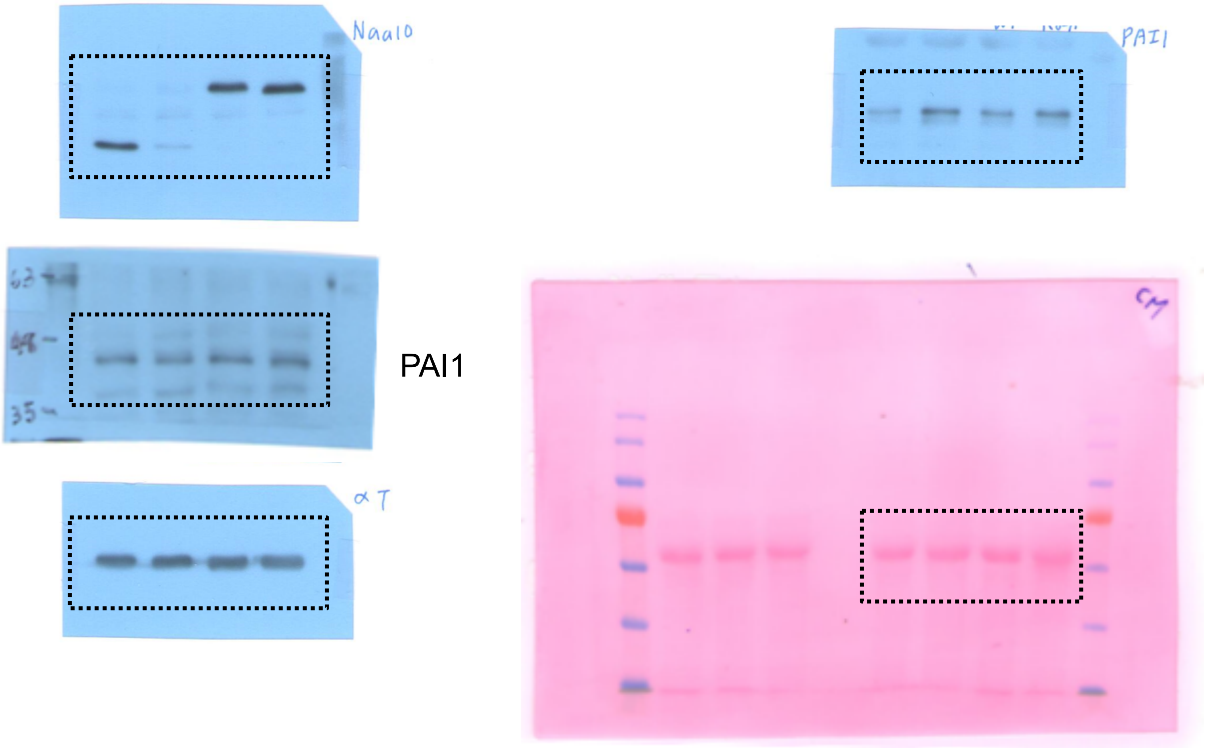
Naa10
PAI1
αT
PAI1

Figure 8.

E

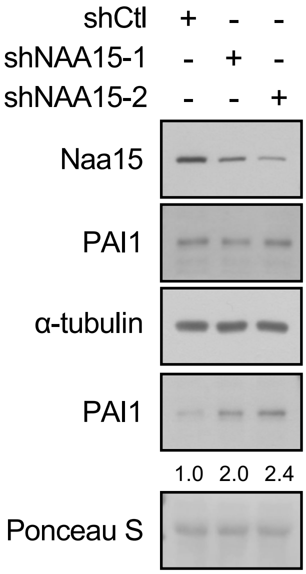

shCtl + - -
shNAA15-1 - + -
shNAA15-2 - - +
Naa15
PAI1
α-tubulin
PAI1
1.0 2.0 2.4
Ponceau S

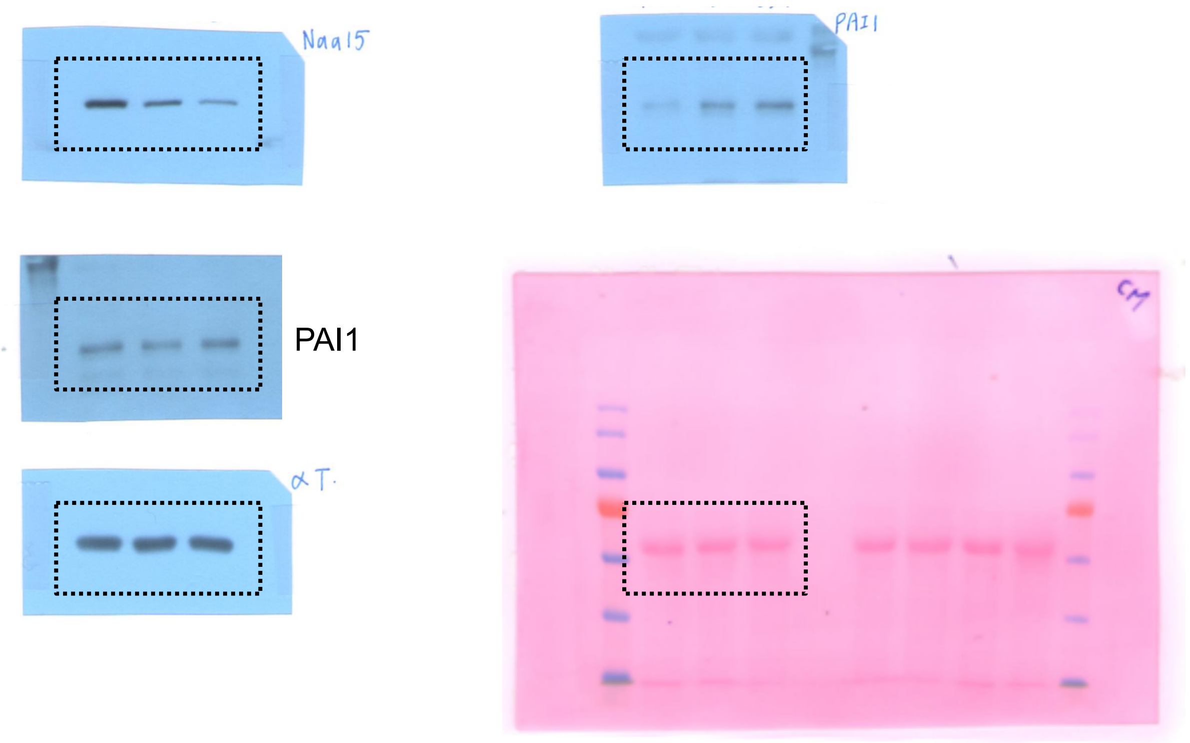

Naa15
PAI1
PAI1
αT.

Figure 8.

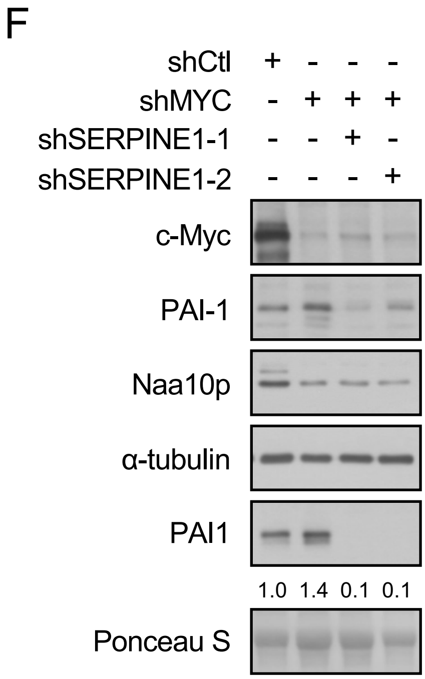
F
shCtl + - - -
shMYC - + + +
shSERPINE1-1 - - + -
shSERPINE1-2 - - - +
c-Myc
PAI-1
Naa10p
α-tubulin
PAI1
1.0 1.4 0.1 0.1
Ponceau S

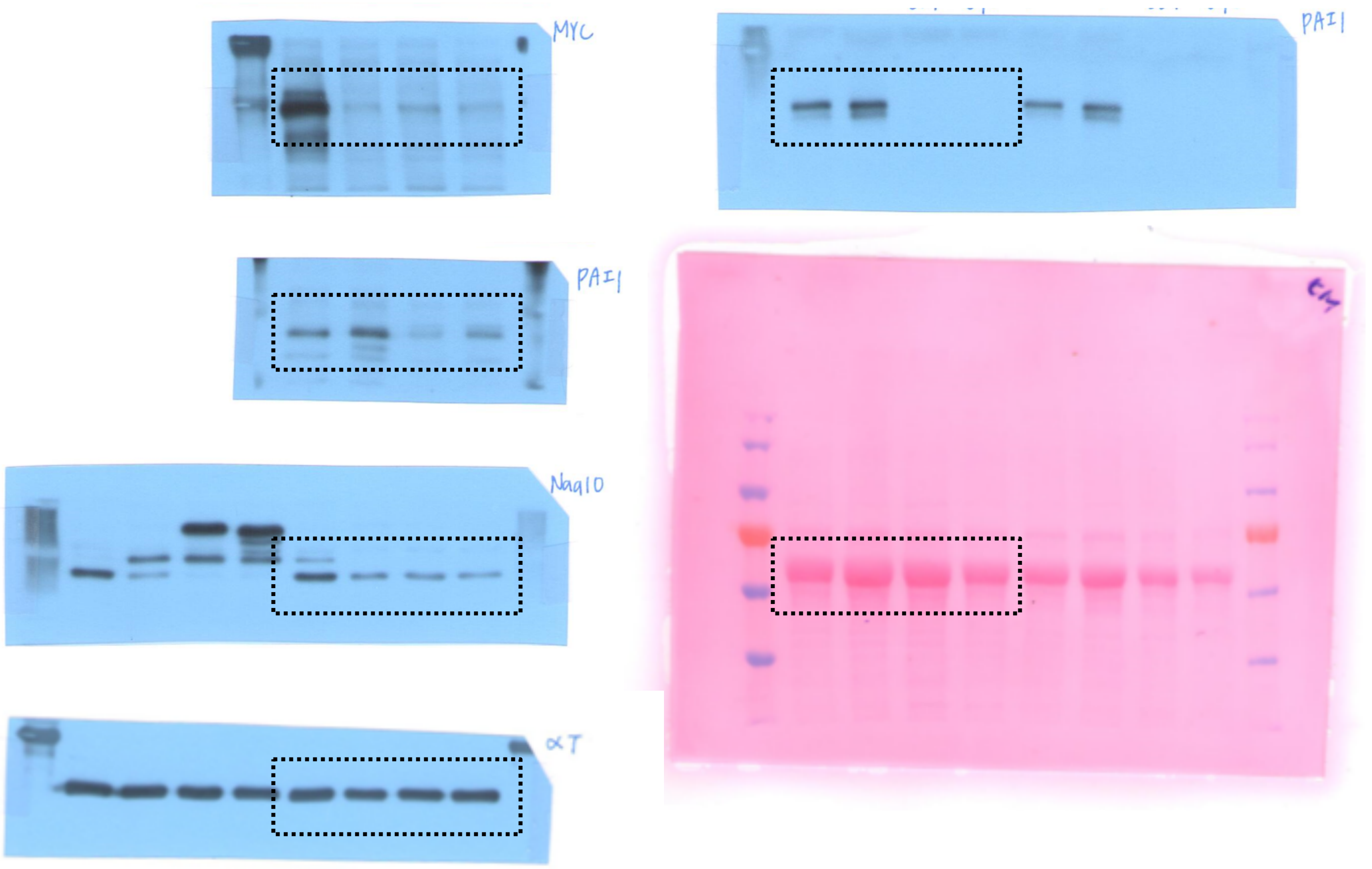
MYC
PAI1
PAI1
Naa10
αT

Figure S2.

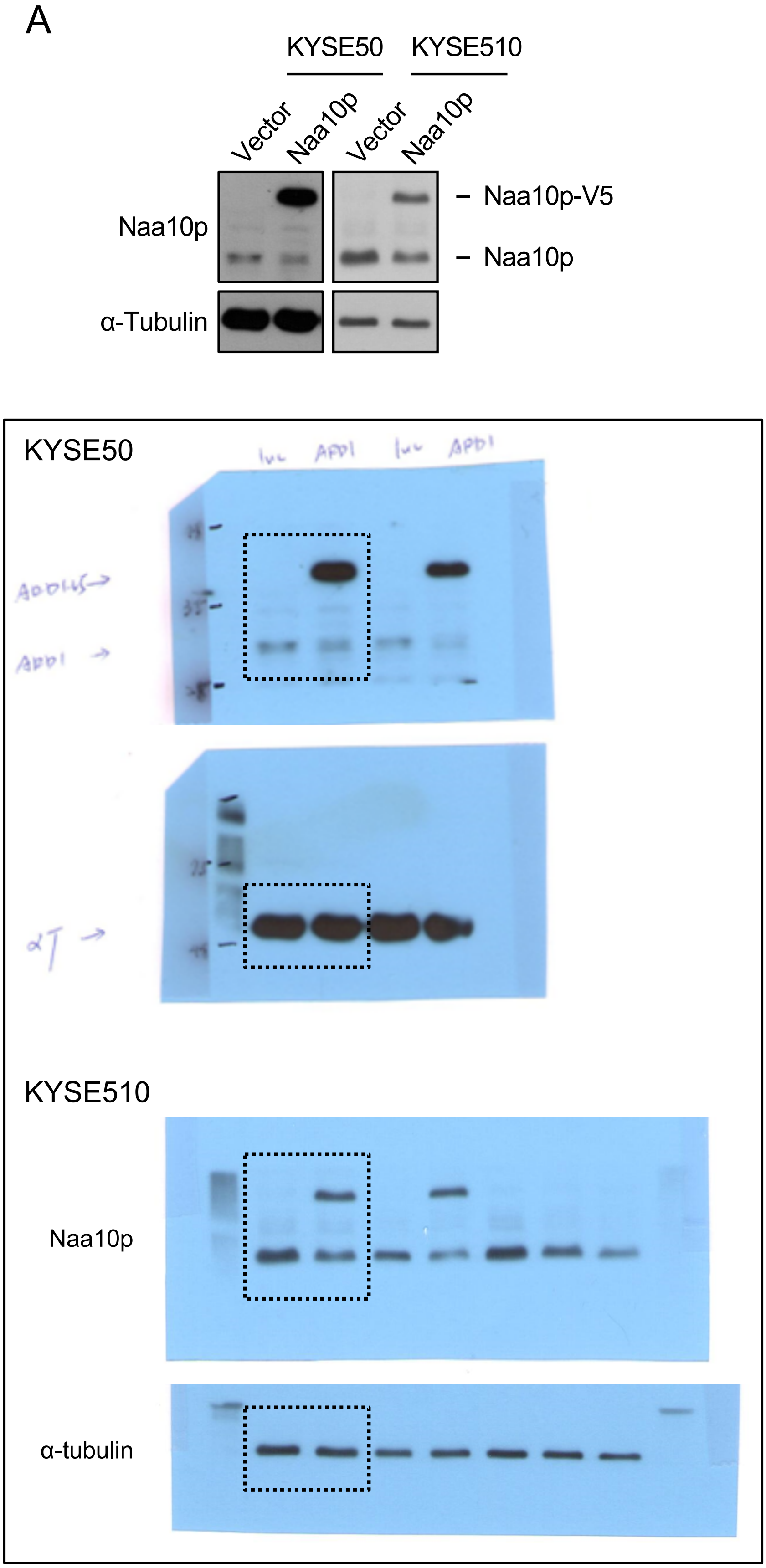

Figure S3.

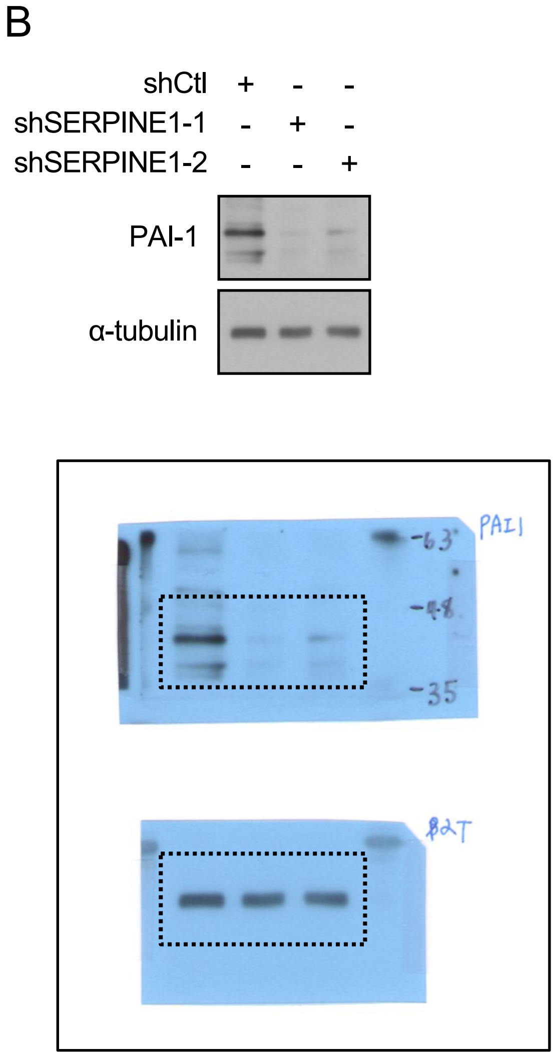

Supplement: Supplementary file 2 — Full and uncropped western blots [file 41419_2022_5441_MOESM2_ESM.pdf]
